# Differential DNA methylation at birth associated with mental disorder in individuals with 22q11.2 deletion syndrome

**DOI:** 10.1038/tp.2017.181

**Published:** 2017-08-29

**Authors:** A Starnawska, C S Hansen, T Sparsø, W Mazin, L Olsen, M Bertalan, A Buil, J Bybjerg-Grauholm, M Bækvad-Hansen, D M Hougaard, P B Mortensen, C B Pedersen, M Nyegaard, T Werge, S Weinsheimer

**Affiliations:** 1 iPSYCH, The Lundbeck Foundation Initiative for Integrative Psychiatric Research, Aarhus, Denmark; 2 Department of Biomedicine, Aarhus University, Aarhus, Denmark; 3 iSEQ, Center for Integrative Sequencing, Aarhus University, Aarhus, Denmark; 4 Section of Neonatal Genetics, Department for Congenital Disorders, Danish Centre for Neonatal Screening, Statens Serum Institute, Copenhagen, Denmark; 5 Institute of Biological Psychiatry, Mental Health Center, Sct. Hans, Mental Health Services, Roskilde, Denmark; 6 Pediatric Oncology Research Laboratory, University Hospital Rigshospitalet, Copenhagen, Denmark; 7 National Centre for Register-Based Research, Aarhus University, Aarhus, Denmark; 8 Centre for Integrated Register-Based Research, Aarhus University, Aarhus, Denmark; 9 Institute of Clinical Sciences, Faculty of Medicine and Health Sciences, University of Copenhagen, Copenhagen, Denmark

## Abstract

Individuals with 22q11.2 deletion syndrome (DS) have an increased risk of comorbid mental disorders including schizophrenia, attention deficit hyperactivity disorder, depression, as well as intellectual disability. Although most 22q11.2 deletion carriers have the long 3-Mb form of the hemizygous deletion, there remains a large variation in the development and progression of psychiatric disorders, which suggests that alternative factors contribute to the pathogenesis. In this study we investigated whether neonatal DNA methylation signatures in individuals with the 22q11.2 deletion associate with mental disorder later in life. DNA methylation was measured genome-wide from neonatal dried blood spots in a cohort of 164 individuals with 22q11.2DS, including 48 individuals diagnosed with a psychiatric disorder. Among several CpG sites with *P*-value<10^−6^, we identified cg23546855 (*P*-value=2.15 × 10^−7^) mapping to *STK32C* to be associated with a later psychiatric diagnosis. Pathway analysis of the top findings resulted in the identification of several Gene Ontology pathways to be significantly enriched (*P*-value<0.05 after Benjamini–Hochberg correction); among them are the following: neurogenesis, neuron development, neuron projection development, astrocyte development, axonogenesis and axon guidance. In addition, we identified differentially methylated CpG sites in *LRP2BP* (*P*-value=5.37 × 10^−8^) to be associated with intellectual disability (F70–79), in *TOP1* (*P*-value=1.86 × 10^−7^) with behavioral disorders (F90–98), in *NOSIP* (*P*-value=5.12 × 10^−8^) with disorders of psychological development (F80–89) and in *SEMA4B* (*P*-value=4.02 × 10^−7^) with schizophrenia spectrum disorders (F20–29). In conclusion, our study suggests an association of DNA methylation differences at birth with development of mental disorder later in life in 22q11.2DS individuals.

## Introduction

There is a strikingly increased risk of developing a mental disorder among individuals diagnosed with 22q11.2 deletion syndrome (DS) including mood disorders, anxiety disorders, attention deficit hyperactivity disorder (ADHD), autism spectrum disorder, as well as intellectual disability (ID) and schizophrenia (SZ).^1, 2, 3, 4, 5, 6, 7^ It is the most common DS in humans that occurs at a population frequency ~1:3700 live births (Olsen *et al.*, manuscript in preparation).^8, 9, 10, 11^ The syndrome is characterized by a hemizygous microdeletion of 1.5–3 Mb of chromosome 22 and is associated with a variety of clinical phenotypes including cardiac defects, hypocalcemia, cleft palate, dysfunction of the immune system as well as multiple mental disorders.^12, 13, 14, 15^

The International Consortium on Brain and Behavior in 22q11.2DS has collectively described the prevalence of psychiatric diagnoses in different age groups based on 15 clinical cohorts, reporting a change in prevalence of mental disorders in 22q11.2DS individuals throughout the lifespan.^1^ In these clinically ascertained cohorts, one-third of carriers have a below-average intellectual level (IQ, 70–84) with adults frequently diagnosed with more severe levels of impairment.^16, 17, 18^ ADHD, particularly the inattentive type, is ~10 times more prevalent in children with the deletion,^19^ with prevalence estimates ranging from 15.6 to 37% across different studies.^1^ The prevalence of autism spectrum disorder among individuals with 22q11.2DS also varies by study/site/instrument used, with estimates ranging from 12.8^1, 4^ to 26.5%,^1^ whereas SZ spectrum disorders and major depression rates reach as high as 41.7 and 15.8%, respectively.^1^ Furthermore, population-based studies suggest that the incidence rates of psychiatric disorders are elevated in clinically identified 22q11.2 deletion carriers relative to the general population.^7, 20^ Notably, odds ratio estimates of 16 or more obtained from genetic association studies^21, 22, 23^ index the 22q11.2 deletion as one of the largest risk factors for SZ spectrum disorder.

Although unbiased population-based disease risk estimates are lacking, the frequency of psychiatric health problems in clinically identified 22q11.2 deletion carriers certainly exceed those of the general population.^7, 20^ Hence, a better understanding of the biological underpinnings for the elevated risk of mental disorders in 22q11.2 deletion carriers will facilitate improved clinical care.

Even though most 22q11.2 deletion carriers have the long 3-Mb form of the deletion, there is a broad variation in the development and progression of psychiatric disorders. This could be driven by other genetic risk factors; however, there is no evidence of the presence of genetic factors on the non-deleted 22q11.2 copy to predispose these individuals to the development of, for example, psychotic disorders.^24, 25, 26^ This suggests that alternative factors, such as environmental and epigenetic factors, could contribute to the development, onset and/or progression of psychiatric phenotypes in 22q11.2DS individuals.

The epigenome is dynamic, changing in early development, throughout life, and even during disease progression. In addition, epigenetic changes have been implicated in the pathophysiology of several psychiatric disorders including SZ, bipolar disorder, major depressive disorder and Rett syndrome.^27, 28, 29, 30, 31, 32, 33, 34, 35^ On the basis of this growing body of evidence, we hypothesized that already at birth the epigenome of individuals with 22q11.2DS who have a psychiatric phenotype differs from those who carry the same deletion but do not have a psychiatric diagnosis. In this study, to our knowledge, we performed the first Epigenome-Wide Association Study (EWAS) in neonatal blood spot samples for a large case cohort of 22q11.2DS individuals, including 29% of individuals with a psychiatric diagnosis. Epigenetic profiles in neonatal blood samples may prove to be a useful tool to identify early on which 22q11.2 deletion carriers are at increased risk for mental disorder, allowing for early prediction and intervention, as well as improved understanding of the pathogenesis that underlies development of psychiatric phenotypes.

## Materials and methods

### Study population

This EWAS study was performed on DNA from neonatal dried blood spot samples collected at birth on filter paper for 164 unrelated 22q11.2DS individuals (80 females and 84 males).

The cohort’s current mean age was 14.7 years (s.d.=6.5). Subjects were selected from The Danish Cytogenetic Central Register, and presence of 22q11.2 deletion was identified via fluorescent *in situ* hybridization and/or comparative genomic hybridization array. Out of all 22q11.2DS cases identified from the Danish Cytogenetic Central Register, 209 individuals had a blood spot sample stored within the Danish Neonatal Screening Biobank (DNSB), which is a part of the Danish National Biobank (those who did not have a blood spot sample were primarily born before 1982). Individuals were excluded from the methylation study if array quality-control failed, genetic validation did not verify the deletion or the deletion mapped downstream of the 22q11.2 locus and hence did not truly represent 22q11.2DS. Type of 22q11.2 deletion was additionally confirmed with genotype data obtained from Infinium PsychArray BeadChip (Illumina, San Diego, CA, USA, data not shown), followed by copy number variant (CNV) prediction using the PennCNV software (http://www.openbioinformatics.org/penncnv/).^36^

Psychiatric diagnosis information was retrieved from the Danish Psychiatric Central Register, which contains information about all admissions to Danish psychiatric inpatient facilities since 4 January 1969 and outpatient since 1995.^37^ In this cohort, a total of 48 individuals (21 females and 27 males) had an ICD-10 level F diagnosis (Mental and Behavioral Disorders), whereas 116 individuals have not had a documented current or past psychiatric diagnosis (called, respectively, psychiatric cases and non-psychiatric controls in the primary analysis). The mean age of individuals with psychiatric diagnosis was 16.7 years (s.d.=6.2).

In secondary analyses, we evaluated the methylomes using further stratification of psychiatric diagnoses in which the psychiatric cohort was subcategorized into four main diagnosis groups: intellectual disability (ID, F70–79: intellectual disability, *n*=23), behavioral disorders (BDs, F90–98: behavioral and emotional disorders with onset usually occurring in childhood and adolescence, *n*=12), disorders of psychological development (PD, F80–89: disorders of psychological development, *n*=16) and SZ spectrum disorders (F20–29: schizotypal and delusional disorders, *n*=4; Supplementary Figure 1). Detailed demographics for this cohort are presented in Supplementary Table 1. Individuals with comorbid mental disorders were included in the analyses as reflected by Supplementary Figure 1.

The study was approved by the Regional Ethical Committee, The Capital Region of Denmark, Copenhagen, Denmark (Arv&Miljø:H-B-2009-026) and Danish Data Protection Agency (PSV-2009-07). Permissions were granted to couple and perform population-based analyzes from the Danish Cytogenetic Central Register, DNSB and Danish National Patient Registry resources (established and maintained according to Danish law). The access is granted to researchers on a case-by-case request by the competent Danish authorities.

### DNA methylation analysis

Genomic DNA was extracted from neonatal dried blood spots at the Danish Neonatal Screening Biobank. For each individual, two disks (each 3.2 mm in diameter) were punched from their dried blood spots stored on Whatman Specimen 903 Collection Paper (GE Healthcare Life Sciences, Chicago, IL, USA). Genomic DNA was extracted with the use of Extract-N-Amp Blood PCR kit (Sigma-Aldrich, St. Louis, MO, USA), without the amplification step. Genomic DNA was bisulfite-converted using the EZ-96 DNA Methylation Kit (Zymo Research, Irvine, CA, USA) according to the manufacturer's instructions. DNA methylation from the bisulfite-converted DNA was measured using the Infinium HumanMethylation450BeadChip array (Illumina) as previously described.^38^

Quality control of DNA methylation data was performed using tools in R-packages ChAMP^39^ and minfi.^40^ Principal components analysis was performed to identify possible outliers. All samples had <5% failed probes. Methylation data were further normalized with the use of within-array Functional Normalization, which regresses out the variability explained by control probes included on the Infinium HumanMethylation450BeadChip array in order to remove the technical variation.^41^ Normalization was performed with the first two principal components.^41^ Normalization was followed by removal of probes with detP>0.01 in at least 20% of samples, cross-reactive probes, probes targeting sex chromosomes and probes known to overlap with common single-nucleotide polymorphisms (SNPs) in the CEU population.^42^ Correction for possible technical batch effects was performed with the Combat tool.^43^ Normalized and batch-corrected methylation values were further logit-transformed.

To determine whether blood cell-composition estimates were different between the study groups, we used the methylation data for estimation of blood cell composition^44^ using the flowSorted.CordBlood.450k reference panel.^45^ This reference panel is based on flow cytometry-sorted cord blood cells for 17 individuals, all of whom contribute between four to seven samples of distinct cell types. We estimated the composition of B cells, CD4 T cells, CD8 T cells, granulocytes, monocytes, natural killer cells and nucleated red blood cells (nRBCs)) for each sample and investigated whether there were significant differences in cell composition between mental disorder, ID, BD, PD, as well as SZ cases and non-psychiatric controls with the use of *t*-test (for normally distributed blood cell estimates) or Mann–Whitney test (for cell types with non-normal distribution).

In addition, to confirm the presence of the 22q11.2 deletion in each individual, we used the DNAcopy package to call CNVs from the methylation data as described before.^46^ On the basis of the regions of CNVs called on chromosome 22, we assessed the boundaries of each deletion type as follows: common LRC22A–LRC22D (18892575–21460000 bp); nested proximal deletion LRC22A–LRC22B (18892575–20330000 bp); nested central deletion LRC22C–LRC22D (20700000–21460000 bp); and distal deletion within LRC22D–LRC22E (21460000–23700000 bp). LRC22A–LRC22B and LRC22C–LRC22D overlap with the LRC22A–LRC22D deletion region, but do not overlap with each other, and the deletion within LRC22D–LCR22E is located downstream of the LRC22A–LRC22D deletion. Boundaries of the 22q11.2 deletion depend on the probe distribution across this region as represented on the methylation array. CNV plots for the 22q11.2 region were visually inspected and CNVs were filtered to include minimum 50 markers with minimum quality of segmentation mean threshold <−0.3 for deletions. The CNV calls were also compared with PennCNV calls for 22q11 region obtained from PsychArray SNP data (Illumina, data not shown).

### Statistical analysis

In this study we used the EWAS approach to determine differential DNA methylation among 22q11.2 deletion carriers diagnosed with mental disorder compared with carriers who do not have any current or past mental disorder diagnosis (non-psychiatric controls; primary analysis). In the secondary analyses, we evaluated four subclasses of most commonly observed psychiatric diagnoses (ID, BD, PD and SZ) in the cohort and compared these profiles to the non-psychiatric controls (same controls for each comparison). We used regression models to identify differentially methylated probes (further referred to as DMP) and included adjustment for sex, type of 22q11.2 deletion and age of the blood spot card to correct for possible storage batch effects. Moreover, we investigated whether the type of 22q11.2 deletion (LRC22A–LRC22D versus LRC22A–LRC22B or LRC22A–LRC22D versus other) associated with altered methylation patterns in the genome (CNV-type association analyses). These analyses were performed with the use of linear regression models, with adjustment for sex and age of the blood spot card. All statistical analyses were performed using R.^47^ In this study we report all DMPs with uncorrected *P*-value<10^−6^ (suggested genome-wide significant threshold for EWAS^48^) and annotated to genes using NCBI build hg19.

### Functional analysis

Pathway analysis was performed on results from each EWAS using genes annotated to DMPs with uncorrected *P*-value<10^−5^. Analyses for enriched Gene Ontology (GO) pathways were performed using the tools available in the WEB-based GEne SeT AnaLysis Toolkit (WEB-Gestalt).^49^ A pathway was considered significant if the Benjamini–Hochberg^50^-corrected *P*-value for pathway enrichment was <0.05. To evaluate underlying genetic networks of the psychiatric-associated genes identified in the EWAS primary analysis (*n*=38 genes, *P*-value<10^−5^), we used the GeneMANIA bioinformatics software with default parameters.^51^ The GeneMANIA algorithm (version 3.4.0, http://www.genemania.org)^52^ utilizes functional interaction data to create a network to illustrate putative functional connections between genes. We used the GeneMANIA Cytoscape 3.2.1 plugin version 3.4.0 (ref. 51) to obtain a network for the psychiatric-associated genes identified in the EWAS primary analysis based on the 2014-08-12 GeneMANIA human database build. This build includes data from known genetic and physical interactions, shared protein domains, coexpression data, as well as primary and curated molecular interaction networks and pathways. The 'query gene-based' weighting method was used in this study in which the top 20 predicted related genes are assigned a score (computed using a variation of Gaussian field label propagation^53, 54^). The score describes the degree of connectivity to the input genes. The network gene pairs are then assigned a weight, which reflects the strength of the interaction.

## Results

### Characterization of the 22q11.2 deletion type

**Figure 1:**
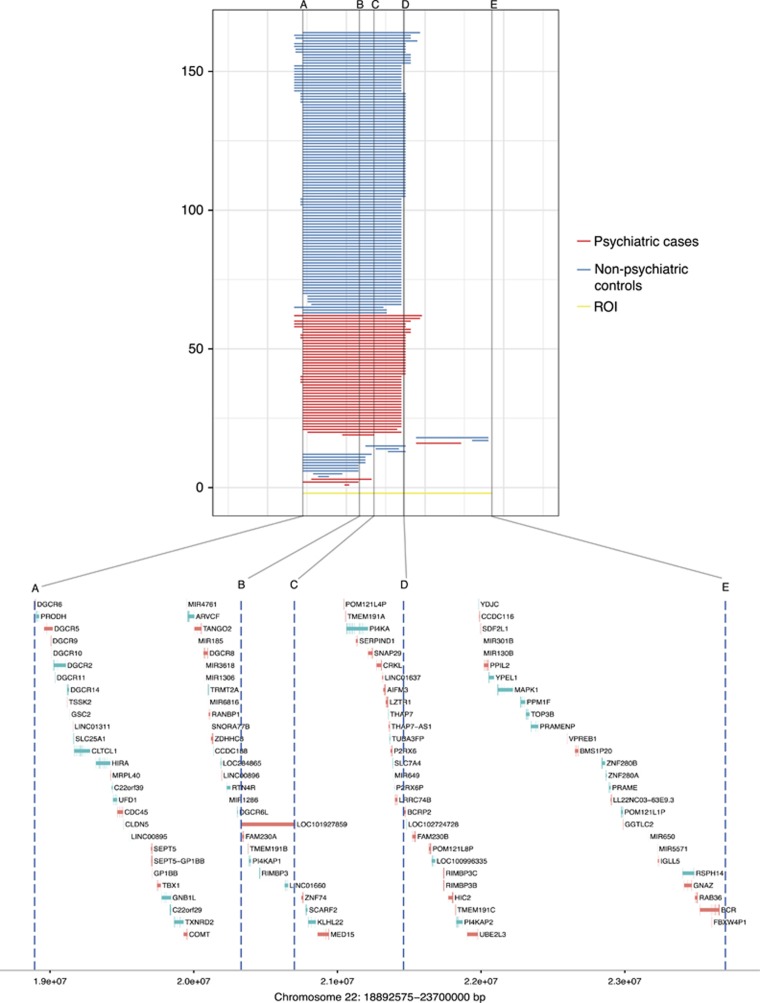
Top panel: Overview of 22q11.2 deletion types obtained by CNV calling from 450 K DNA methylation array for all 164 individuals included in the sample arranged by 22q11.2 deletion type. Horizontal gray lines marked A–E indicates boundaries of different 22q11.2 deletion types. ROI indicates 22q11.2 deletion region of interest as defined by genotyping data for these individuals. Bottom panel: Overview of RefSeq genes (hg19) located in the region of 22q11.2 deletion. Orange color indicates genes transcribed from the sense strand; blue color indicates genes transcribed from the antisense strand. CNV, copy number variant.

In this cohort we observed four types of 22q11.2 deletions characterized by length and position (Figure 1). A total of 146 (~89%) individuals were found to carry the LRC22A–LRC22D deletion, 12 (~7%) had LRC22A–LRC22B type, 6 (~4%) individuals carried a deletion within the LRC22C–LRC22D or LRC22D–LCR22E region (Figure 1). Among 48 individuals with psychiatric diagnosis, 44 (~92%) had LRC22A–LRC22D deletion, 4 (~8%) had deletion within LRC22A–LRC22B or LRC22D–LCR22E region of 22q11.2, and none had LRC22C–LRC22D deletion. These results were concordant with PennCNV calls in SNP genotype data (data not shown) and support the use of 450 K DNA methylation data for detection of known CNVs. All samples passed quality-control and probe quality and filtering steps resulted in 420 363 probes that were included in the regression analyses.

### Estimation of blood cell composition

No significant differences (*P*-value>0.05) were found for B cells, CD4 T cells, CD8 T cells, granulocytes, monocytes and natural killer cell composition between investigated groups. The percentage of nRBC in our sample was estimated to be extremely low (median nRBC=0.016, with 1st quantile 0.0079 and 3rd quantile 0.024), which was expected, given these cells are present in cord blood, but steadily decline to zero within the first week of life.^55^ Because of the similarity of blood cell composition across the study groups, the estimates were not added as covariates to the regression models.

### Primary analysis: EWAS for psychiatric phenotype among 22q11.2 deletion carriers

**Figure 2:**
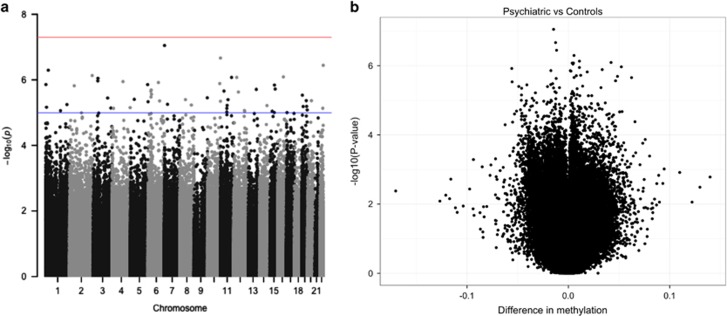
Overview of signals and corresponding effect sizes for results obtained from EWAS of 22q11.2DS individuals with psychiatric phenotype versus controls. Each dot represents one probe used in the EWAS analysis. (**a**) Manhattan plot depicting significance levels for all probes included in the primary analysis (blue line marks −log10(1e−05), red line marks −log10(5e−08) significance level); (**b**) volcano plot depicting effect sizes compared with the significance levels of all probes included in the primary analysis. DS, deletion syndrome; EWAS, Epigenome-Wide Association Study.

**Figure 3:**
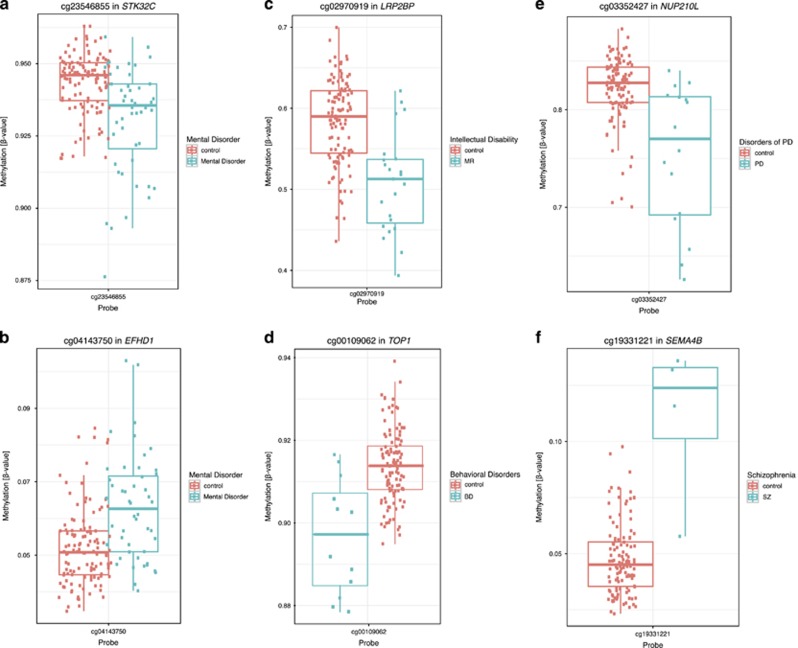
Overview of DNA methylation values for the highly associated CpG sites in EWAS of psychiatric diagnosis, site in *STKC32* (*P*-value=2.15 × 10^−7^; **a**) and in *EFHD1* (*P*-value=7.39 × 10^−7^; **b**); intellectual disability, site in *LRP2BP* (*P*-value=5.37 × 10^−8^; **c**); behavioral disorders, site in *TOP1* (*P*-value=1.86 × 10^−7^; **d**); disorders of psychological development, site in *NUP210L* (*P*-value=1.67 × 10^−8^; **e**); schizophrenia, site in *SEMA4B* (*P*-value=4.02 × 10^−7^; **f**). EWAS, Epigenome-Wide Association Study.

We first compared the methylome between deletion carriers who had developed a mental disorder to deletion carriers with no current or past diagnosis of mental disorder (22q11.2 non-psychiatric controls). This primary analysis identified nine DMPs with *P*-value<10^−6^ (Table 1 and Figure 2). In all, 22.2% of these DMPs were located in gene bodies, 33.3% in intergenic regions (IGRs), 44.4% in a region up to 1500 bp upstream from transcription start site (TSS) in comparison with 33.5% in bodies, 23.2% in IGRs and 25.9% in up to 1500 bp TSS of probes included for the EWAS analysis. The most significantly associated DMPs mapped to six genes: hypomethylation in Serine/Threonine Kinase 32C (*STK32C*), hypermethylation in WD and tetratricopeptide repeats 1 (*WDTC1*), EF-Hand Domain Family, Member D1 (*EFHD1*), nucleoporin 93 (*NUP93*), neural cell adhesion molecule 1 (*NCAM1*) and zinc-finger MYND-type containing 10 (*ZMYND10*). The remaining three associated DM probes were intergenic, including the top associated hypomethylated DMP cg21509978 (*P*-value=8.91 × 10^−8^) located in a DNaseI hypersensitivity region on chromosome 7, located 23.6 kb away from the gene family with sequence similarity member C (*FAM20C*; Table 1). Boxplots for the probes associated in *STK32C* and *EFHD1* are presented as Figures 3a and b.

GO pathway analysis identified 10 significantly enriched pathways (Benjamini–Hochberg-adjusted *P*-value<0.05), including neurogenesis, neuron development, astrocyte development and axon guidance (Supplementary Table 2). Psychiatric-associated DMPs (*P*-value<10^−5^) were annotated to a total of 38 genes, which were input to GeneMANIA to identify genetic networks. The network analysis illustrates that 36 of the 38 genes were connected in a complex interactive network with a high degree of coexpression and several genes with many genetic interactions, for example, *STK32C* and *NUP210L* (Supplementary Figure 2).

### Secondary analyses: EWAS of psychiatric subphenotypes

In an exploratory analysis, the 48 individuals with a psychiatric disorder were further characterized by psychiatric subclassification and secondary EWAS were performed to identify DMPs for the four most abundant diagnoses (ID, BD, PD and SZ), summarized in Table 1 and Figure 3. Nine DMPs were associated with ID including the most significant DMP mapping to Low Density Lipoprotein Receptor-Related Protein 2 Binding Protein (*LRP2BP*, Figure 3c). Two DMPs were associated with BD, one of which mapped to Type I DNA Topoisomerase (*TOP1*) and the other to an IGR on chromosome 14 (Figure 3d).

Eight DMPs associated with PD with the top associated DMPs mapping to Nucleoporin 210 kDa-Like (*NUP210L*) and Nucleoporin 93kDa (*NUP93*; Figure 3e). Two DMPs located 9 bp apart mapped to Nitric Oxide Synthase Interacting Protein (*NOSIP*; Supplementary Figure 4).

Three DMPs were associated with SZ, including two in known genes, Heat Shock Factor Binding Protein 1 (*HSBP1*) and Sema Domain, Immunoglobulin Domain, Transmembrane Domain And Short Cytoplasmic Domain (Semaphorin), 4B (*SEMA4B*), whereas the other maps to an IGR of chromosome 4 (Figure 3f).

There was no overlap of significant DMPs between the subpsychiatric diagnoses; however, several DMPs overlapped across the diagnoses when considering *P*-value<10^−5^ (Supplementary Figure 5). GO pathway analysis for the ID DMPs identified enrichment in pathways involved in myoblast differentiation. There were no GO pathways significantly enriched in BD, PD or SZ EWAS analyses.

### CNV-type association analysis

**Figure 4:**
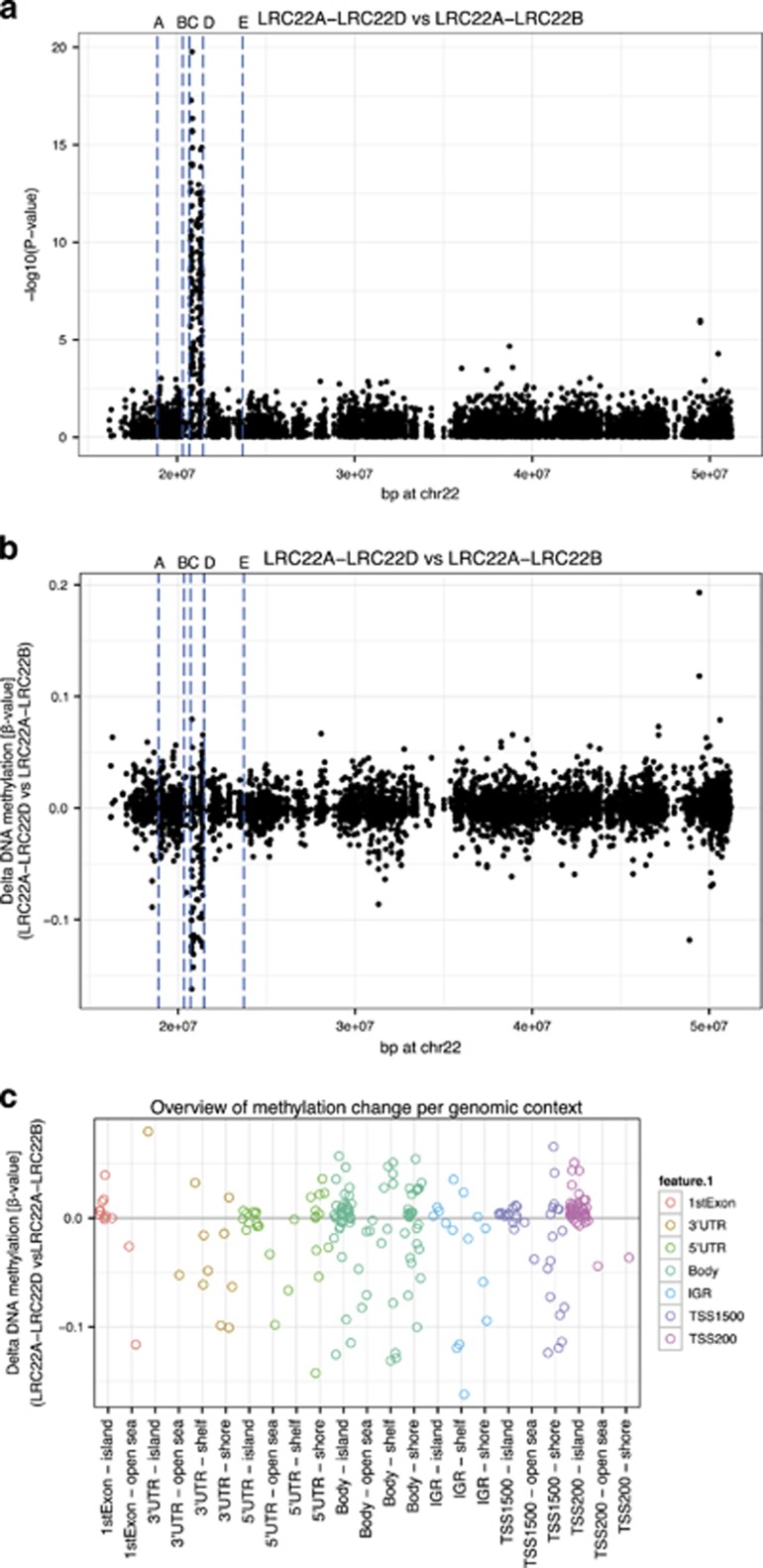
Overview of results located on chromosome 22 from CNV EWAS comparing methylome profiles of 22q11.2 cases with LRC22A–LRC22D versus LRC22A–LRC22B deletion type. Overview of *P*-values is presented in **a**, effect sizes in **b** and genomic and CGI context of findings associated in LRC22C–LRC22D region in **c**. Horizontal blue lines on **a**, **b** indicate boundaries of different 22q11.2 deletion types (lines A–D: LRC22A–LRC22D; A and B: LRC22A–LRC22B; C and D: LRC22C–LRC22D; D and E: LRC22D–LRC22E). CNV, copy number variant; EWAS, Epigenome-Wide Association Study.

Gene dosage may influence DNA methylation either locally at the CNV or elsewhere in the genome; hence, we evaluated whether the specific type of 22q11.2 deletion associates with a distinctive DNA methylation signature independent of the psychiatric diagnosis. We identified 107 DMPs in the comparison of methylomes between the LRC22A–LRC22D and LRC22A–LRC22B deletion, 100 of which were located on chromosome 22 (Supplementary Table 3) and mapped to 16 known genes at the LRC22C–LRC22D deletion region (Figure 4a, located in boundaries C–D) including Zinc Finger Protein 74 (*ZNF74*), Scavenger Receptor Class F, Member 2 (*SCARF2*), Mediator Complex Subunit 15 (*MED15*), Synaptosomal-Associated Protein, 29 kDa (*SNAP29*) and Apoptosis-Inducing Factor, Mitochondrion-Associated, 3 (*AIFM3*).

Effect sizes of the findings located on chromosome 22, presented as difference of the mean methylation between individuals with LRC22A–LRC22D and those with LRC22A–LRC22B deletion, are presented at Figure 4b. Most of the DMPs mapping to chromosome 22 were within 200 bp of the TSS (200TSS) or in the gene body, and were located in a CpG Island (Figure 4c). The distribution of 107 DMPs in TSS and IGRs was different than expected by random with 31.8% of DMPs located in TSS200 in comparison with only 11.4% of probes targeting this genomic feature in this EWAS. In turn, 7.5% DMPs were located in IGRs in comparison with 23.2% of probes targeting this genomic feature. No other striking differences were observed in the distribution of 107 DMPs across other genomic features.

A total of 11.2% of DMPs (versus 14.5% probed) were located in TSS1500, 32.7% of DMPs (versus 33.5% probed) were in gene bodies, 7.5% DMPs (versus 9.0% probed) were in 5′ untranslated regions (UTRs), 4.7% DMPs (versus 3.5% probed) were in 3′ UTRs and 4.7% DMPs (versus 4.8% probed) were in the first exon. Five of the seven additional DMPs for LRC22A–LRC22D versus LRC22A–LRC22B CNV type mapped to the following loci: chromosome 5 open reading frame 38 (*C5orf38*), N-acyl phosphatidylethanolamine phospholipase D (*NAPEPLD*), tubulin alpha 3c (*TUBA3C*) and cg20496134 in Family With Sequence Similarity 128B (*FAM128B*), also known as Mitotic Spindle Organizing Protein 2B (*MZT2B*), and located 4668 bp upstream cg10992590 located in the same intron of *MZT2B*, but also overlapping with Sphingomyelin Phosphodiesterase 4, Neutral Membrane (Neutral Sphingomyelinase-3; *SMPD4*; Supplementary Figures 6A–G).

Comparison of the LRC22A–LRC22D deletion with all other deletion types resulted in 112 DMPs (Supplementary Table 4); the majority of these (*n*=99) overlapped with the DMPs identified from LRC22A–LRC22D versus LRC22A–LRC22B analysis. Overlap of these DMPs was expected, as the majority of deletions present in the ‘other’ category are the LRC22A–LRC22B type, which is driving the differential methylation signal. After addition of other types of CNV to the sample, we observed an increase in significance level of DMPs located in LRC22A–LRC22B and decrease of signal in the LRC22C–LRC22D region.

## Discussion

Epigenetic studies performed in samples collected at birth have the potential to reveal neonatal predictors of mental disorders before manifestation of their clinical symptoms. In this study, to our knowledge, we performed the first EWAS in a large cohort of individuals with 22q11.2DS and report that differential DNA methylation in neonatal blood spots is associated with mental disorders manifested later in life. After careful inspection of methylation data and application of standard quality-control measures, we found the neonatal blood spots to be a useful resource to evaluate the whole-genome DNA methylation profiles, and also confirm the 22q11.2 deletion for each individual in the cohort as documented in the Danish Cytogenetic Central Register. Even though the estimation of the deletion boundaries depended on the probe distribution on the methylation array, we observed that the boundaries assessed independently by methylation array and genotyping array gave very comparable results, thus supporting CNV calling from array-based methylome data as previously proposed.^46^ Distribution of different 22q11.2 deletion types was comparable between those with mental disorder and those without, with one exception that LRC22C–LRC22D deletion was not observed among individuals with diagnosed mental disorders. However, since only three individuals harbor the LRC22C–LRC22D deletion, larger studies of 22q11.2DS will be able to provide evidence whether this deletion type influences risk of mental disorders. Frequencies of 22q11.2 deletion subtypes observed in this cohort were comparable to previously published estimates.^56^

We identified nine DMPs at birth that were differentially methylated between 22q11.2DS individuals with a psychiatric diagnosis and those without a psychiatric diagnosis. Several of the DMPs map to genes that have been previously implicated in mental disorders. For example, we observed hypomethylation of a CpG site in an intron of *STK32C* in individuals with a psychiatric diagnosis. A CpG site in *STK32C* located 31 235 bp from the one we report here was previously reported to be associated with adolescent depression and major depressive disorder in a recent methylome study of buccal cells from monozygotic twins.^33^ The signal was additionally replicated in post-mortem cerebellum tissue from an independent cohort of cases suffering from major depressive disorder and unaffected individuals.^33^ Interestingly, 22q11.2DS individuals are known to have increased risk of developing major depressive disorder, with prevalence at adolescence estimated to reach 9%.^1^ Thus, the differential methylation detected at birth in *STK32C* may contribute to the risk of mental disorder for 22q11.2DS individuals. *STK32C* is known to be highly expressed in the human brain, with highest expression levels reported in the cerebellum and frontal cortex.^57^

Interestingly, a recent study confirmed that DNA methylation across *STK32C* is highly predictive of cell-type-specific (neuronal versus glial cells) gene expression profiles.^58^ Thus, even though the specific function of this kinase is still unknown, it can be speculated that it has a role in cellular differentiation or cell-type specificity in the brain. In addition, using the Blood Brain DNA Methylation Comparison Tool (http://epigenetics.iop.kcl.ac.uk/bloodbrain/),^59^ which has information on DNA methylation in blood and four brain regions, we found that the CpG site of interest (cg23546855) in *STK32C* is highly methylated in cerebellum samples and blood, which was consistent with results in our study (mean methylation of cg23546855=0.94 in our sample). However, even though cg23546855 is highly methylated in both cerebellum and blood, it needs to be further investigated if methylation level of this site also co-varies between these tissues and how it relates to brain gene expression levels.

We also observed hypomethylation of *EFHD1* to be associated with mental disorder. Expression levels of *EFHD1* are progressively increasing in the cerebrum and cerebellum during neuronal differentiation in mouse postnatal development.^60^ Interestingly, *EFHD1* is among 108 loci significantly associated with SZ in a large genome-wide association study performed in 36 989 SZ cases and 113 075 controls.^61^ *EFHD1* was among other genes driving the signal for significant pathway enrichment analyses, where neurogenesis and neuron projection development GO pathways were found to be significantly enriched. Since 22q11.2DS is associated with changes in brain volume and widespread alterations in brain anatomy, identification of differential methylation of genes involved in neurogenesis and neuronal projection can provide a putative mechanism for these alterations.^62^ In addition, several other genes that function in these pathways were differentially methylated: glial cell-derived neurotrophic factor (*GDNF*), stress-induced-phosphoprotein 1 (*STIP1*) and neural cell adhesion molecule 1 (*NCAM1*), which has a well-established role in neurodevelopment and synaptic plasticity.^63, 64, 65^ Interestingly, a recent SZ study reported hypomethylation of *NCAM1* in a post-mortem prefrontal cortex to be the most disorder-associated finding.^66^ These results point to a potential link between DNA methylation of genes involved in neuronal development and mental disorder diagnosis later in life. Indeed, several genes in our study function in pathways involved in neurodevelopment and neurogenesis, which supports previous studies that suggested that mental disorders have an early neurodevelopmental component and methylomic abnormalities may be responsible for these effects.^35, 67, 68^

It is unknown whether the DNA methylation differences observed in this study of birth blood samples will remain stable over time until the onset of disorder, as methylation is dynamic throughout development.^69, 70^ In a recent study that investigated the epigenetic profiles of ADHD symptom trajectories,^71^ differential methylation signals observed at birth in blood were no longer significantly associated with ADHD trajectories at the age of 7. These results suggest that some epigenetic signatures may be detectable only at specific developmental stages. On the basis of the study by Walton *et al.*,^71^ we could hypothesize that the birth DNA methylation signatures reported in our manuscript may be unique for this developmental stage and reflect only early changes in the methylome that contribute to risk of developing a psychiatric disorder. Future 22q11 studies including a longitudinal collection of blood (for example, at birth, at diagnosis and at follow-up) will facilitate the identification of methylation signatures present at birth that remain stable risk markers for mental illness.

In this study we also performed analyses of subphenotypes of mental disorders in the 22q11.2DS cohort. We observed significant hypomethylation of the CpG site in the 1500TSS region of the *LRP2BP* gene in individuals with ID. *LRP2BP* is most highly expressed in the pituitary gland, as well as in cerebellum, and acts as an adapter that regulates LRP2 receptor function.^57, 72^ Mutations in the *LRP2* gene have been associated with forebrain malformations,^73^ autism spectrum disorder^74^ and mild ID.^75^ Moreover, genetic mutations in the *LRP2* gene are known to cause Donnai–Barrow syndrome^76^ characterized by agenesis of the corpus callosum, developmental delay, as well as mild to moderate ID.^77^

Analysis of BDs led to the identification of differential methylation in the 1500TSS region of *TOP1*, an enzyme responsible for altering the topology of DNA.^78^ Inhibition of *TOP1* in mouse neurons results in decreased expression of exceptionally long genes, caused by impaired transcription elongation, many of which are known to be autism spectrum disorder candidate genes.^79^

In EWAS of disorders of psychological development we observed hypomethylation of two probes located in a CpG island of *NOSIP*, which is critical for brain and craniofacial development in mice and is a candidate gene for SZ^61^ and holoprosencephaly, a common developmental disorder in humans.^80^ In addition, *NOSIP* interacts and colocalizes with neuronal nitric oxide synthase (*nNOS*) to regulate its distribution and activity.^81^ Knockout of *nNOS* in mice results in a striking increase in behavioral abnormalities including aggressive and excess sexual behaviors.^82^ *NOSIP* and *nNOS* are concentrated in neuronal synapses and overexpression of *NOSIP* was shown to reduce the availability of *nNOS* in terminal dendrites.^81^ Hence, our observations support a role for *NOSIP* regulation in psychological developmental disorders.

Despite the fact that this 22q11.2 cohort is relatively young and only four individuals have been diagnosed with SZ, we observed hypermethylation at a CpG site located in the 200TSS region of *SEMA4B* associated with this phenotype. The gene has low expression levels in adult brain;^57^ however, Sema4B is reported to have a crucial role in glutamatergic and GABAergic synaptic development in cultured hippocampal neurons, which may contribute to SZ.^83^

In this study we also considered the influence of 22q11.2 deletion type on the methylation landscape and observed a gene-dosage effect on the methylation profile specifically at the local 22q11.2 region. Interestingly, 93.5% of the DM probes (*P*-value<10^−6^) associated with CNV type (LRC22A–LRC22D versus LRC22A–LRC22B) mapped to the region on chromosome 22 where the LRC22C–LRC22D deletion occurs. LRC22C–LRC22D is the region on chromosome 22 that distinguishes LRC22A–LRC22B deletion type from the LRC22A–LRC22D. As individuals carrying the hemizygous LRC22A–LRC22D deletions are missing one copy of all genes in the region, there appears to be a gene-dosage effect on the methylation profile specifically at the local 22q11.2 region.

The effect of CNV on DNA methylation has not been well studied. However, Strong *et al.*^84^ reported a similar observation with symmetrical dose-dependent DNA methylation profile in children with deletion or duplication of 7q11.23. We observed that DMPs from this analysis are over-represented in 200TSS and under-represented in IGRs. In addition, when we characterized differential methylation between the LRC22A–LRC22D versus all other 22q11.2 deletion types, there was a marked decrease in the number of DM probes at the LRC22C–LRC22D site and an increase in the DM probes at the LRC22A–LRC22B deletion site. Thus, even with limited number of individuals in this cohort who carry LRC22D–LRC22E, we detect differences that enable us to speculate that CNVs may cause a local shift in the methylome pattern. This may be a compensatory mechanism for the loss of gene dosage at the site. Future studies including more individuals who carry the atypical 22q11.2 CNVs and non-22q11.2DS controls will be needed to elucidate the biological effect of CNV on local methylome patterns.

Moreover, seven additional CpG sites not located on chromosome 22 were associated with the 22q11.2 deletion type, two of which mapped to *MZT2B* on chromosome 2 and *NAPEPLD* on chromosome 7. *MZT2B* encodes a protein essential for mitotic spindle assembly by centrosomal recruitment of γ-tubulin.^85^ *NAPEPLD* encodes an enzyme that catalyzes formation of N-acylethanolamines utilized as signaling molecules in the nervous system,^86^ and is primarily expressed in the brain, especially in the cerebral hemisphere and cerebellum.^57^

Although the models used in this study included adjustment for potential confounders such as blood spot storage time, sex and 22q11.2 deletion type, this study has several limitations. First, methylation patterns are tissue-specific; thus, the methylation differences observed in blood spots at birth may serve as a marker for phenotypic risk but may not reflect the brain methylome, nor define the biological development and/or progression to mental disorder. DNA methylation patterns are also influenced by blood cell composition and gestational age, as reported in umbilical cord blood studies.^87^ Despite the fact that none of the DMPs reported in this study overlapped with genes reported to associate with gestational age,^87^ it should be noted that the HumanMethylation27 BeadChip array (Illumina) may be not robust enough to identify all genes associated with gestational age. In addition, birth complications can result in negative outcomes that increase the risk of mental disorders,^88^ as well as impact the epigenome.^89^ However, information on pregnancy and birth complications was unavailable for our cohort; thus, future studies will be necessary to assess the relation between birth complications, DNA methylation signatures and mental disorders.

In addition, although 29% of the individuals in this 22q11.2 cohort have a diagnosed mental disorder, more are likely to experience future onset of mental illness. For example, in our sample only 2% of individuals have been diagnosed with SZ. The cohort is quite young and many have not yet reached the mean age of SZ onset; thus, we expect more cases to develop the disorder later in life and future analyses shall include re-evaluation of the adult cohort.^3, 90, 91^ In addition, because of sampling bias and differences in information collection methods, it is noteworthy that the psychiatric case group in this study is likely to represent the more severe cases of mental disorders and these phenotypes may be discrepant from other reports. For example, the percentage of cases with ID (14%) was relatively low in comparison with the reported 51% in the literature.^4^ We obtained psychiatric diagnosis information from the Danish Psychiatric Central Register, which is based on hospitalization record, whereas in the study by Niklasson *et al.* the severity of ID was extracted from neuropsychiatric assessments and questionnaires.^4^ In addition, because of the high heterogeneity of mental disorders among 22q11.2 cases, dissection of the cohort into the most common psychiatric phenotypes led to a very small sample size for these secondary analyses, which reduced the power to identify differential methylation, and could lead to possible identification of false-positives. With this limitation, results from the secondary analyses should be considered as preliminary; however, further collection of neonatal samples from 22q11.2 deletion carriers will allow for an increase in sample size and statistical power and will permit validation of our findings in an independent cohort

In conclusion, we report evidence of genome-wide significant methylation differences for mental disorder in 22q11.2DS. The genes were enriched with those involved in neurogenesis, nervous development and neuron projection development. We also detected alterations in genome-wide DNA methylation patterns associated with the type of 22q11.2 deletion. Our data suggest that altered DNA methylation measured at birth may be a useful approach to identify genes that contribute to the pathophysiology of mental disorders. Future epigenetic studies of an independent 22q11.2DS cohort will be required to replicate these EWAS findings and longitudinal studies would complement this research to identify early epigenetic biomarkers for mental disorders.

## Figures and Tables

**Table 1:** Overview of findings with *P*-value<10^−6^ obtained from EWAS analysis of psychiatric phenotypes in individuals with 22q11.2DS

	*Probe ID*	*P-value*	*CHR*	*bp*	*Gene*	*Genomic feature*
Psychiatric (*n*=48) versus controls (*n*=116)	cg21509978	8.91E−08	7	169 560	NA	IGR—open sea
	cg23546855	2.15E−07	10	134 068 039	*STK32C*	Body—shelf
	cg22877639	3.59E−07	22	51 023 896	NA	IGR—shelf
	cg01396034	5.07E−07	1	27 623 367	*WDTC1*	Body—open sea
	cg04143750	7.39E−07	2	233 497 656	*EFHD1*	TSS1500—shore
	cg09308553	8.09E−07	16	56 763 573	*NUP93*	TSS1500—shore
	ch.12.31424680R	8.39E−07	12	31 533 413	NA	IGR—open sea
	cg02126412	8.40E−07	11	112 831 938	*NCAM1*	TSS200—shore
	cg04322105	8.97E−07	3	50 383 309	*ZMYND10*	TSS200—island
						
Intellectual disability (*n*=23) versus controls (*n*=116)	cg02970919	5.37E−08	4	186 300 945	*LRP2BP*	TSS1500—open sea
	cg25743026	1.30E−07	6	107 194 450	NA	IGR—open sea
	cg08540622	3.41E−07	11	59 806 898	*PLAC1L*	TSS1500—open sea
	cg22052566	3.68E−07	7	151 079 167	*WDR86*	Body—island
	cg07941927	6.13E−07	13	100 644 041	NA	IGR—shore
	cg07186765	7.05E−07	7	30 633 504	*GARS*	TSS1500—shore
	cg11571263	7.56E−07	6	90 121 836	*RRAGD*	5′ UTR—island
	cg24062754	7.87E−07	5	175 965 158	*RNF44*	TSS1500—shore
	cg06597895	8.00E−07	3	44 935 097	*TGM4*	Body—open sea
						
Behavioral disorders (*n*=12) versus controls (*n*=116)	cg01000937	7.77E−08	14	30 730 636	NA	IGR—open sea
	cg00109062	1.86E−07	20	39 655 980	*TOP1*	TSS1500—shore
						
Disorders of psychological development (*n*=16) versus controls (*n*=116)	cg03352427	1.67E−08	1	154 127 523	*NUP210L*	1stExon—open sea
	cg09308553	6.00E−08	16	56 763 573	*NUP93*	TSS1500—shore
	cg01764082	1.43E−07	9	98 784 014	*NCRNA 00092*	Body—island
	cg04355077	5.12E−07	19	50 059 946	*NOSIP*	Body—island
	cg22489957	8.26E−07	6	28 921 805	NA	IGR—open sea
	cg19696083	8.50E−07	12	54 438 419	*HOXC4*	5′ UTR—shelf
	cg21292909	8.86E−07	19	50 059 937	*NOSIP*	Body—island
	cg08427305	9.99E−07	19	30 165 133	*PLEKHF1*	Body—island
						
Schizophrenia (*n*=4) versus controls (*n*=116)	cg12815697	1.19E−20	16	83 841 917	*HSBP1*	Body—island
	cg13298070	1.55E−10	4	189 580 507	NA	IGR—island
	cg19331221	4.02E−07	15	90 728 027	*SEMA4B*	TSS200—island

Abbreviations: 1stExon, first exon of the gene; 5′ UTR, 5′ untranslated region; Body, gene body; CHR, chromosome; DS, deletion syndrome; EWAS, Epigenome-Wide Association Study; IGR, intergenic region; island, CpG island; open sea, >4 kb up- or downstream from CpG island; NA, not available (no gene maps to this CpG site); shelf, 2–4 kb up- or downstream from CpG island; shore, 0–2 kb up- or downstream from CpG island; TSS, transcription start site (200—up to 200 bp upstream from TSS,1500—up to 1500 bp upstream from TSS).

Annotation based on UCSC (genome.ucsc.edu) GRCh37/hg19 reference.
