# Supplementary material for: Differential DNA methylation at birth associated with mental disorder in individuals with 22q11.2 deletion syndrome
Source: Transl Psychiatry. 2017 Aug 29;7(8):e1221–. doi: 10.1038/tp.2017.181 (PMC5611746; doi:10.1038/tp.2017.181)
Supplement: Supplementary Table 1 [file tp2017181x1.docx]

Supplementary Table 1. Demographics for individuals with 22q11.2 DS.

|  | **Cohort** | | | | | |
| --- | --- | --- | --- | --- | --- | --- |
|  | **Psychiatric**  **(n=48)** | **Control**  **(n=116)** | **Intellectual Disability**  **(n=23)** | **Psychological Development**  **(n=16)** | **Behavioral Disorders**  **(n=12)** | **SZ**  **(n=4)** |
| Age (years)  Mean ± SD  Range | 16.65 (6.18)  4 – 29 | 13.94 (6.46)  3 – 28 | 18.35 (4.31)  9 – 29 | 16.5 (5.57)  8 – 29 | 15.17 (4.47)  6 – 21 | 20.25 (na)  na |
| Sex  % Male | 56.3 | 49.1 | 52.2 | 75 | 75 | na |

Abbreviation: SD, standard deviation; SZ, schizophrenia; na, sample size too small to maintain anonymity
