# Supplementary material for: Differential DNA methylation at birth associated with mental disorder in individuals with 22q11.2 deletion syndrome
Source: Transl Psychiatry. 2017 Aug 29;7(8):e1221–. doi: 10.1038/tp.2017.181 (PMC5611746; doi:10.1038/tp.2017.181)
Supplement: Supplementary Table 2 [file tp2017181x2.docx]

| **GO Pathway Name** | **GO Pathway ID** | **Pathway Statistics** |
| --- | --- | --- |
| cell development | ID:GO:0048468 | C=1431; O=11; E=3.13; R=3.52; rawP=0.0001; adjP=0.0171 |
| neurogenesis | ID:GO:0022008 | C=1120; O=10; E=2.45; R=4.09; rawP=8.27e-05; adjP=0.0171 |
| Golgi apparatus | ID:GO:0005794 | C=1128; O=9; E=2.51; R=3.59; rawP=0.0006; adjP=0.0360 |
| astrocyte development | ID:GO:0014002 | C=13; O=2; E=0.03; R=70.42; rawP=0.0004; adjP=0.0457 |
| axon guidance | ID:GO:0007411 | C=355; O=5; E=0.78; R=6.45; rawP=0.0009; adjP=0.0495 |
| cell morphogenesis involved in neuron differentiation | ID:GO:0048667 | C=570; O=6; E=1.25; R=4.82; rawP=0.0013; adjP=0.0495 |
| neuron projection morphogenesis | ID:GO:0048812 | C=576; O=6; E=1.26; R=4.77; rawP=0.0013; adjP=0.0495 |
| neuron development | ID:GO:0048666 | C=783; O=7; E=1.71; R=4.09; rawP=0.0012; adjP=0.0495 |
| axonogenesis | ID:GO:0007409 | C=521; O=6; E=1.14; R=5.27; rawP=0.0008; adjP=0.0495 |
| neuron projection development | ID:GO:0031175 | C=690; O=7; E=1.51; R=4.64; rawP=0.0006; adjP=0.0495 |

Supplementary Table 2. Overview of significant results from GO analysis for enriched pathways (BH adjusted p-value < 0.05) obtained from primary EWAS analysis of psychiatric phenotype among individuals with 22q11.2 DS.

C: the number of reference genes in the category;

O: the number of genes in the gene set and also in the category;

E: the expected number in the category;

R: ratio of enrichment;

rawP: p value from hypergeometric test;

adjP: p value adjusted by the multiple test adjustment
