# Supplementary material for: Differential DNA methylation at birth associated with mental disorder in individuals with 22q11.2 deletion syndrome
Source: Transl Psychiatry. 2017 Aug 29;7(8):e1221–. doi: 10.1038/tp.2017.181 (PMC5611746; doi:10.1038/tp.2017.181)
Supplement: Supplementary Table 3 [file tp2017181x3.docx]

Supplementary Table 3. Overview of findings at p-value < 10^-6^ obtained from EWAS analysis of LRC22A-LRC22D vs LRC22A-LRC22B deletion in individuals with 22q11.2 DS.

| **Probe ID** | **P-value** | **CHR** | **Bp** | **arm** | **Gene** | **Genomic feature** |
| --- | --- | --- | --- | --- | --- | --- |
| cg21448991 | 1.72E-20 | 22 | 20850176 | q | *KLHL22* | TSS200 - island |
| cg15243570 | 5.32E-18 | 22 | 20792217 | q | *SCARF2* | TSS200 - island |
| cg27035678 | 4.43E-17 | 22 | 20861940 | q | *MED15* | 1stExon - island |
| cg03894033 | 1.86E-16 | 22 | 20850168 | q | *KLHL22* | TSS200 - island |
| cg14722674 | 2.11E-16 | 22 | 20861944 | q | *MED15* | 1stExon - island |
| cg27645955 | 1.39E-15 | 22 | 21386885 | q | *SLC7A4* | TSS200 - island |
| cg04369837 | 1.43E-15 | 22 | 20861720 | q | *MED15* | TSS200 - island |
| cg16590005 | 1.82E-15 | 22 | 21319245 | q | *AIFM3* | TSS200 - island |
| cg25714069 | 9.32E-15 | 22 | 20791214 | q | *SCARF2* | Body - island |
| cg02014809 | 9.92E-15 | 22 | 20861768 | q | *MED15* | TSS200 - island |
| cg26796825 | 1.18E-14 | 22 | 20861479 | q | *MED15* | TSS1500 - shore |
| cg19518452 | 1.40E-14 | 22 | 21336528 | q | *LZTR1* | TSS200 - island |
| cg22320035 | 9.36E-14 | 22 | 20764816 | q | NA | IGR - shelf |
| cg10768682 | 1.12E-13 | 22 | 21213158 | q | *SNAP29* | TSS200 - island |
| cg14998335 | 2.07E-13 | 22 | 20792255 | q | *SCARF2* | TSS200 - island |
| cg07841312 | 2.36E-13 | 22 | 20748432 | q | *ZNF74* | TSS200 - island |
| cg24333469 | 2.59E-13 | 22 | 21386894 | q | *SLC7A4* | TSS200 - island |
| cg04088817 | 2.62E-13 | 22 | 21311408 | q | NA | IGR - island |
| cg06659169 | 2.62E-13 | 22 | 20748430 | q | *ZNF74* | TSS200 - island |
| cg06658625 | 3.12E-13 | 22 | 21133553 | q | *SERPIND1* | 5'UTR - open sea |
| cg20750843 | 3.31E-13 | 22 | 21271406 | q | *CRKL* | TSS1500 - island |
| cg07685736 | 6.46E-13 | 22 | 21320014 | q | *AIFM3* | 5'UTR - shore |
| cg05684406 | 6.75E-13 | 22 | 21386914 | q | *SLC7A4* | TSS200 - island |
| cg19990744 | 1.08E-12 | 22 | 21333817 | q | *AIFM3* | Body - shelf |
| cg12082202 | 1.20E-12 | 22 | 21356477 | q | *FLJ39582* | Body - island |
| cg16469441 | 1.34E-12 | 22 | 20850308 | q | *KLHL22* | TSS200 - island |
| cg03952331 | 1.45E-12 | 22 | 20792222 | q | *SCARF2* | TSS200 - island |
| cg00500213 | 3.02E-12 | 22 | 21337671 | q | *LZTR1* | Body - shore |
| cg25418528 | 3.84E-12 | 22 | 21381565 | q | *P2RX6* | 3'UTR - open sea |
| cg06985578 | 5.54E-12 | 22 | 21333706 | q | *AIFM3* | Body - shelf |
| cg14107273 | 5.84E-12 | 22 | 21271661 | q | *CRKL* | TSS200 - island |
| cg08615820 | 6.49E-12 | 22 | 21210457 | q | *PI4KA* | 5'UTR - shelf |
| cg00831466 | 8.02E-12 | 22 | 20795872 | q | *KLHL22* | 3'UTR - shelf |
| cg03143742 | 8.04E-12 | 22 | 20792143 | q | *SCARF2* | 5'UTR - island |
| cg26919527 | 8.36E-12 | 22 | 21133973 | q | *PI4KA* | Body - open sea |
| cg10956413 | 1.33E-11 | 22 | 21057317 | q | *TMEM191A* | Body - island |
| cg00051704 | 1.35E-11 | 22 | 21353809 | q | NA | IGR - shore |
| cg10568796 | 1.71E-11 | 22 | 21193857 | q | *PI4KA* | 5'UTR - open sea |
| cg08788246 | 2.52E-11 | 22 | 20758901 | q | *ZNF74* | Body - shore |
| cg14271231 | 4.60E-11 | 22 | 20759325 | q | *ZNF74* | Body - shore |
| cg19711553 | 4.70E-11 | 22 | 20850288 | q | *KLHL22* | TSS200 - island |
| cg20401551 | 5.08E-11 | 22 | 20790985 | q | *SCARF2* | Body - island |
| cg19508107 | 6.75E-11 | 22 | 21335080 | q | *AIFM3* | Body - shore |
| cg08140634 | 6.84E-11 | 22 | 21356518 | q | *FLJ39582* | Body - island |
| cg21244955 | 7.36E-11 | 22 | 21192955 | q | *PI4KA* | Body - open sea |
| cg14835423 | 7.76E-11 | 22 | 20748341 | q | *ZNF74* | TSS200 - island |
| cg14096051 | 9.68E-11 | 22 | 21356040 | q | *FLJ39582* | TSS200 - island |
| cg11558591 | 9.90E-11 | 22 | 21356472 | q | *FLJ39582* | Body - island |
| cg23001415 | 1.17E-10 | 22 | 21311401 | q | NA | IGR - island |
| cg22507989 | 1.78E-10 | 22 | 21356069 | q | *FLJ39582* | TSS200 - island |
| cg03448766 | 1.96E-10 | 22 | 20792243 | q | *SCARF2* | TSS200 - island |
| cg06685464 | 2.63E-10 | 22 | 20790837 | q | *SCARF2* | Body - island |
| cg18393023 | 3.08E-10 | 22 | 21126967 | q | *SERPIND1* | TSS1500 - open sea |
| cg11511562 | 3.78E-10 | 22 | 21359962 | q | *FLJ39582* | Body - shelf |
| cg21014483 | 3.79E-10 | 22 | 21368707 | q | *P2RX6* | TSS1500 - island |
| cg00411011 | 3.90E-10 | 22 | 20848478 | q | *KLHL22* | 5'UTR - shore |
| cg25976804 | 4.92E-10 | 22 | 21335632 | q | *AIFM3* | 3'UTR - shore |
| cg19840066 | 5.07E-10 | 22 | 20792224 | q | *SCARF2* | TSS200 - island |
| cg16500810 | 7.99E-10 | 22 | 21271516 | q | *CRKL* | TSS200 - island |
| cg09788239 | 9.27E-10 | 22 | 20862292 | q | *MED15* | Body - shore |
| cg11982252 | 1.23E-09 | 22 | 21350285 | q | *LZTR1* | Body - shelf |
| cg13813874 | 1.78E-09 | 22 | 20849595 | q | *KLHL22* | 5'UTR - island |
| cg09481857 | 1.82E-09 | 22 | 21368659 | q | *P2RX6* | TSS1500 - island |
| cg15773198 | 1.92E-09 | 22 | 20760502 | q | *ZNF74* | Body - island |
| cg26919378 | 1.94E-09 | 22 | 20791300 | q | *SCARF2* | Body - island |
| cg19265040 | 2.15E-09 | 22 | 20861470 | q | *MED15* | TSS1500 - shore |
| cg05471139 | 2.49E-09 | 22 | 20748332 | q | *ZNF74* | TSS200 - island |
| cg20100936 | 2.58E-09 | 22 | 21271664 | q | *CRKL* | TSS200 - island |
| cg06144260 | 2.74E-09 | 22 | 21397511 | q | *P2RX6P* | Body - shelf |
| cg20758953 | 2.87E-09 | 22 | 20762620 | q | *ZNF74* | 3'UTR - shore |
| cg20180721 | 4.33E-09 | 22 | 21213387 | q | *SNAP29* | 1stExon - island |
| cg14831990 | 4.74E-09 | 22 | 21400167 | q | *LOC400891* | TSS200 - island |
| cg26290543 | 6.66E-09 | 11 | 45377324 | p | NA | IGR - shore |
| cg03733278 | 6.73E-09 | 22 | 20760922 | q | *ZNF74* | Body - island |
| cg17353431 | 8.41E-09 | 22 | 21356784 | q | *FLJ39582* | Body - island |
| cg22628623 | 9.29E-09 | 22 | 21213081 | q | *PI4KA* | TSS200 - island |
| cg01462546 | 9.52E-09 | 22 | 20864312 | q | *MED15* | Body - shelf |
| cg22807537 | 1.27E-08 | 22 | 20851436 | q | *KLHL22* | TSS1500 - shore |
| cg05168344 | 1.36E-08 | 22 | 21340160 | q | *LZTR1* | Body - shelf |
| cg16934981 | 1.67E-08 | 22 | 21336404 | q | *LZTR1* | TSS200 - island |
| cg12874479 | 2.00E-08 | 22 | 21028739 | q | NA | IGR - shelf |
| cg06912515 | 2.13E-08 | 22 | 21368603 | q | *P2RX6* | TSS1500 - island |
| cg20496134 | 2.79E-08 | 2 | 130945155 | q | *FAM128B* | Body - open sea |
| cg09634469 | 2.97E-08 | 22 | 21128411 | q | *SERPIND1* | 1stExon - open sea |
| cg00534362 | 3.40E-08 | 22 | 21335304 | q | *AIFM3* | Body - shore |
| cg12977146 | 3.48E-08 | 22 | 20850163 | q | *KLHL22* | TSS200 - island |
| cg23699239 | 3.71E-08 | 22 | 21356776 | q | *FLJ39582* | Body - island |
| cg10348922 | 3.84E-08 | 22 | 21319303 | q | *AIFM3* | TSS200 - island |
| cg26608332 | 4.57E-08 | 22 | 20861476 | q | *MED15* | TSS1500 - shore |
| cg11888151 | 5.11E-08 | 22 | 21272339 | q | *CRKL* | 1stExon - island |
| cg13096307 | 7.04E-08 | 22 | 20747254 | q | *ZNF74* | TSS1500 - shore |
| cg08159594 | 7.14E-08 | 7 | 102790168 | q | *NAPEPLD* | TSS1500 - shore |
| cg13720744 | 8.21E-08 | 22 | 21271392 | q | *CRKL* | TSS1500 - island |
| cg20350943 | 1.04E-07 | 22 | 20810155 | q | *KLHL22* | Body - open sea |
| cg10992590 | 1.45E-07 | 2 | 130940487 | q | *SMPD4* | TSS200 - shore |
| cg24828246 | 1.51E-07 | 13 | 19749816 | q | *TUBA3C* | Body - shore |
| cg00958955 | 1.54E-07 | 22 | 20752751 | q | *ZNF74* | Body - shelf |
| cg10071880 | 1.98E-07 | 22 | 21268466 | q | NA | IGR - shelf |
| cg02523400 | 2.27E-07 | 22 | 21128191 | q | *SERPIND1* | TSS200 - open sea |
| cg09978259 | 2.61E-07 | 22 | 21352343 | q | *LZTR1* | 3'UTR - shore |
| cg13778336 | 2.81E-07 | 1 | 13836752 | p | NA | IGR - shelf |
| cg23860217 | 3.10E-07 | 22 | 21356466 | q | *FLJ39582* | Body - island |
| cg01657694 | 3.36E-07 | 22 | 20849950 | q | *KLHL22* | 5'UTR - island |
| cg20646217 | 4.54E-07 | 22 | 21356059 | q | *FLJ39582* | TSS200 - island |
| cg10958362 | 6.41E-07 | 5 | 2754016 | p | *C5orf38* | Body - island |
| cg17239761 | 7.48E-07 | 22 | 21213665 | q | *SNAP29* | Body - island |
| cg25785378 | 8.41E-07 | 22 | 20862326 | q | *MED15* | Body - shore |

5’UTR: 5’ Untranslated Region; TSS: Transcription Start Site (200 – up to 200 bp upstream from TSS,1500 – up to 1500 bp upstream from TSS); 1stExon: First exon of the gene; Body: gene body; IGR: Intergenic Region; island: CpG island; shore: 0-2kb up- or downstream from CpG island; shelf: 2-4kb up- or downstream from CpG island; open sea: > 4 kb up- or downstream from CpG island. Annotation based on UCSC (genome.ucsc.edu) GRCh37/hg19 reference.
