# Supplementary material for: Differential DNA methylation at birth associated with mental disorder in individuals with 22q11.2 deletion syndrome
Source: Transl Psychiatry. 2017 Aug 29;7(8):e1221–. doi: 10.1038/tp.2017.181 (PMC5611746; doi:10.1038/tp.2017.181)
Supplement: Supplementary Table 4 [file tp2017181x4.docx]

Supplementary Table 4. Overview of findings at p-value < 10^-6^ obtained from EWAS analysis of LRC22A-LRC22D vs other types of deletion in individuals with 22q11.2 DS.

| **CpG** | **P-value** | **CHR** | **Bp** | **arm** | **Gene** | **Genomic feature** |
| --- | --- | --- | --- | --- | --- | --- |
| cg21448991 | 8.36E-18 | 22 | 20850176 | q | *KLHL22* | TSS200 - island |
| cg25714069 | 3.43E-17 | 22 | 20791214 | q | *SCARF2* | Body - island |
| cg14722674 | 3.13E-16 | 22 | 20861944 | q | *MED15* | 1stExon - island |
| cg27035678 | 5.49E-16 | 22 | 20861940 | q | *MED15* | 1stExon - island |
| cg15243570 | 6.17E-16 | 22 | 20792217 | q | *SCARF2* | TSS200 - island |
| cg04369837 | 1.11E-15 | 22 | 20861720 | q | *MED15* | TSS200 - island |
| cg00831466 | 3.79E-15 | 22 | 20795872 | q | *KLHL22* | 3'UTR - shelf |
| cg00500213 | 5.38E-15 | 22 | 21337671 | q | *LZTR1* | Body - shore |
| cg03894033 | 7.43E-15 | 22 | 20850168 | q | *KLHL22* | TSS200 - island |
| cg16590005 | 8.18E-15 | 22 | 21319245 | q | *AIFM3* | TSS200 - island |
| cg22320035 | 3.35E-14 | 22 | 20764816 | q | NA | IGR - shelf |
| cg26796825 | 3.57E-14 | 22 | 20861479 | q | *MED15* | TSS1500 - shore |
| cg14998335 | 1.17E-13 | 22 | 20792255 | q | *SCARF2* | TSS200 - island |
| cg19518452 | 1.95E-13 | 22 | 21336528 | q | *LZTR1* | TSS200 - island |
| cg07841312 | 2.55E-13 | 22 | 20748432 | q | *ZNF74* | TSS200 - island |
| cg24333469 | 2.57E-13 | 22 | 21386894 | q | *SLC7A4* | TSS200 - island |
| cg14271231 | 3.20E-13 | 22 | 20759325 | q | *ZNF74* | Body - shore |
| cg25418528 | 3.72E-13 | 22 | 21381565 | q | *P2RX6* | 3'UTR - open sea |
| cg20758953 | 5.67E-13 | 22 | 20762620 | q | *ZNF74* | 3'UTR - shore |
| cg00051704 | 6.24E-13 | 22 | 21353809 | q | NA | IGR - shore |
| cg00411011 | 6.39E-13 | 22 | 20848478 | q | *KLHL22* | 5'UTR - shore |
| cg03952331 | 7.23E-13 | 22 | 20792222 | q | *SCARF2* | TSS200 - island |
| cg27645955 | 1.16E-12 | 22 | 21386885 | q | *SLC7A4* | TSS200 - island |
| cg06658625 | 1.18E-12 | 22 | 21133553 | q | *SERPIND1* | 5'UTR - open sea |
| cg26919527 | 1.34E-12 | 22 | 21133973 | q | *PI4KA* | Body - open sea |
| cg08788246 | 1.42E-12 | 22 | 20758901 | q | *ZNF74* | Body - shore |
| cg05684406 | 1.82E-12 | 22 | 21386914 | q | *SLC7A4* | TSS200 - island |
| cg02014809 | 2.00E-12 | 22 | 20861768 | q | *MED15* | TSS200 - island |
| cg06659169 | 4.34E-12 | 22 | 20748430 | q | *ZNF74* | TSS200 - island |
| cg06144260 | 4.41E-12 | 22 | 21397511 | q | *P2RX6P* | Body - shelf |
| cg14096051 | 5.22E-12 | 22 | 21356040 | q | *FLJ39582* | TSS200 - island |
| cg19265040 | 9.24E-12 | 22 | 20861470 | q | *MED15* | TSS1500 - shore |
| cg21014483 | 9.79E-12 | 22 | 21368707 | q | *P2RX6* | TSS1500 - island |
| cg12082202 | 1.15E-11 | 22 | 21356477 | q | *FLJ39582* | Body - island |
| cg08140634 | 1.22E-11 | 22 | 21356518 | q | *FLJ39582* | Body - island |
| cg09481857 | 1.67E-11 | 22 | 21368659 | q | *P2RX6* | TSS1500 - island |
| cg20401551 | 1.71E-11 | 22 | 20790985 | q | *SCARF2* | Body - island |
| cg11511562 | 1.94E-11 | 22 | 21359962 | q | *FLJ39582* | Body - shelf |
| cg13813874 | 5.26E-11 | 22 | 20849595 | q | *KLHL22* | 5'UTR - island |
| cg16500810 | 6.30E-11 | 22 | 21271516 | q | *CRKL* | TSS200 - island |
| cg14107273 | 8.12E-11 | 22 | 21271661 | q | *CRKL* | TSS200 - island |
| cg09788239 | 8.80E-11 | 22 | 20862292 | q | *MED15* | Body - shore |
| cg03143742 | 9.35E-11 | 22 | 20792143 | q | *SCARF2* | 5'UTR - island |
| cg20750843 | 9.35E-11 | 22 | 21271406 | q | *CRKL* | TSS1500 - island |
| cg10768682 | 9.71E-11 | 22 | 21213158 | q | *SNAP29* | TSS200 - island |
| cg14831990 | 9.99E-11 | 22 | 21400167 | q | *LOC400891* | TSS200 - island |
| cg19990744 | 1.15E-10 | 22 | 21333817 | q | *AIFM3* | Body - shelf |
| cg19840066 | 1.26E-10 | 22 | 20792224 | q | *SCARF2* | TSS200 - island |
| cg21244955 | 1.36E-10 | 22 | 21192955 | q | *PI4KA* | Body - open sea |
| cg19711553 | 1.47E-10 | 22 | 20850288 | q | *KLHL22* | TSS200 - island |
| cg16469441 | 1.63E-10 | 22 | 20850308 | q | *KLHL22* | TSS200 - island |
| cg06685464 | 1.76E-10 | 22 | 20790837 | q | *SCARF2* | Body - island |
| cg10956413 | 2.02E-10 | 22 | 21057317 | q | *TMEM191A* | Body - island |
| cg12874479 | 2.40E-10 | 22 | 21028739 | q | NA | IGR - shelf |
| cg26290543 | 2.55E-10 | 11 | 45377324 | p | NA | IGR - shore |
| cg00534362 | 2.56E-10 | 22 | 21335304 | q | *AIFM3* | Body - shore |
| cg17353431 | 2.67E-10 | 22 | 21356784 | q | *FLJ39582* | Body - island |
| cg07685736 | 2.80E-10 | 22 | 21320014 | q | *AIFM3* | 5'UTR - shore |
| cg15773198 | 4.05E-10 | 22 | 20760502 | q | *ZNF74* | Body - island |
| cg03733278 | 4.38E-10 | 22 | 20760922 | q | *ZNF74* | Body - island |
| cg18393023 | 4.50E-10 | 22 | 21126967 | q | *SERPIND1* | TSS1500 - open sea |
| cg10568796 | 6.28E-10 | 22 | 21193857 | q | *PI4KA* | 5'UTR - open sea |
| cg03448766 | 6.53E-10 | 22 | 20792243 | q | *SCARF2* | TSS200 - island |
| cg22507989 | 7.46E-10 | 22 | 21356069 | q | *FLJ39582* | TSS200 - island |
| cg11558591 | 1.04E-09 | 22 | 21356472 | q | *FLJ39582* | Body - island |
| cg19508107 | 1.06E-09 | 22 | 21335080 | q | *AIFM3* | Body - shore |
| cg25976804 | 1.07E-09 | 22 | 21335632 | q | *AIFM3* | 3'UTR - shore |
| cg08615820 | 1.08E-09 | 22 | 21210457 | q | *PI4KA* | 5'UTR - shelf |
| cg23699239 | 1.37E-09 | 22 | 21356776 | q | *FLJ39582* | Body - island |
| cg05471139 | 1.46E-09 | 22 | 20748332 | q | *ZNF74* | TSS200 - island |
| cg22807537 | 1.61E-09 | 22 | 20851436 | q | *KLHL22* | TSS1500 - shore |
| cg05168344 | 1.72E-09 | 22 | 21340160 | q | *LZTR1* | Body - shelf |
| cg09634469 | 2.90E-09 | 22 | 21128411 | q | *SERPIND1* | 1stExon - open sea |
| cg01462546 | 2.93E-09 | 22 | 20864312 | q | *MED15* | Body - shelf |
| cg04088817 | 3.23E-09 | 22 | 21311408 | q | NA | IGR - island |
| cg10071880 | 3.79E-09 | 22 | 21268466 | q | NA | IGR - shelf |
| cg06985578 | 4.02E-09 | 22 | 21333706 | q | *AIFM3* | Body - shelf |
| cg26919378 | 5.10E-09 | 22 | 20791300 | q | *SCARF2* | Body - island |
| cg01419881 | 6.80E-09 | 22 | 19037065 | q | *DGCR11* | TSS1500 - open sea |
| cg14835423 | 9.56E-09 | 22 | 20748341 | q | *ZNF74* | TSS200 - island |
| cg20350943 | 9.98E-09 | 22 | 20810155 | q | *KLHL22* | Body - open sea |
| cg11982252 | 1.15E-08 | 22 | 21350285 | q | *LZTR1* | Body - shelf |
| cg13096307 | 1.21E-08 | 22 | 20747254 | q | *ZNF74* | TSS1500 - shore |
| cg09978259 | 1.34E-08 | 22 | 21352343 | q | *LZTR1* | 3'UTR - shore |
| cg14559799 | 1.44E-08 | 22 | 20760090 | q | *ZNF74* | Body - island |
| cg02157633 | 1.45E-08 | 22 | 19035933 | q | *DGCR2* | Body - open sea |
| cg00958955 | 1.46E-08 | 22 | 20752751 | q | *ZNF74* | Body - shelf |
| cg12977146 | 2.45E-08 | 22 | 20850163 | q | *KLHL22* | TSS200 - island |
| cg02523400 | 2.85E-08 | 22 | 21128191 | q | *SERPIND1* | TSS200 - open sea |
| cg11888151 | 3.08E-08 | 22 | 21272339 | q | *CRKL* | 1stExon - island |
| cg20100936 | 3.25E-08 | 22 | 21271664 | q | *CRKL* | TSS200 - island |
| cg16934981 | 3.40E-08 | 22 | 21336404 | q | *LZTR1* | TSS200 - island |
| cg26608332 | 3.49E-08 | 22 | 20861476 | q | *MED15* | TSS1500 - shore |
| cg20180721 | 5.92E-08 | 22 | 21213387 | q | *SNAP29* | 1stExon - island |
| cg20496134 | 9.07E-08 | 2 | 130945155 | q | *FAM128B* | Body - open sea |
| cg18094551 | 1.06E-07 | 22 | 20004364 | q | *ARVCF* | TSS200 - island |
| cg13720744 | 1.10E-07 | 22 | 21271392 | q | *CRKL* | TSS1500 - island |
| cg23001415 | 1.47E-07 | 22 | 21311401 | q | NA | IGR - island |
| cg22628623 | 1.91E-07 | 22 | 21213081 | q | *PI4KA* | TSS200 - island |
| cg08159594 | 2.13E-07 | 7 | 102790168 | q | *NAPEPLD* | TSS1500 - shore |
| cg25577379 | 2.31E-07 | 22 | 20939103 | q | *MED15* | Body - island |
| cg06135725 | 3.12E-07 | 22 | 20307624 | q | *DGCR6L* | TSS200 - island |
| cg10992590 | 3.39E-07 | 2 | 130940487 | q | *SMPD4* | TSS200 - shore |
| cg14481263 | 4.51E-07 | 22 | 20008608 | q | *C22orf25* | TSS200 - island |
| cg10614909 | 6.90E-07 | 22 | 20105024 | q | *TRMT2A* | TSS1500 - island |
| cg17239761 | 6.95E-07 | 22 | 21213665 | q | *SNAP29* | Body - island |
| cg12391945 | 7.06E-07 | 22 | 19515608 | q | NA | IGR - shelf |
| cg00604193 | 7.31E-07 | 22 | 19035893 | q | *DGCR2* | Body - open sea |
| cg06942142 | 8.20E-07 | 22 | 21307595 | q | *CRKL* | 3'UTR - shelf |
| cg16322302 | 9.00E-07 | 3 | 49157769 | p | *USP19* | 5'UTR - island |
| cg00399938 | 9.65E-07 | 22 | 20118646 | q | *ZDHHC8* | TSS1500 - island |
| cg25785378 | 9.89E-07 | 22 | 20862326 | q | *MED15* | Body - shore |

5’UTR: 5’ Untranslated Region; TSS: Transcription Start Site (200 – up to 200 bp upstream from TSS,1500 – up to 1500 bp upstream from TSS); 1stExon: First exon of the gene; Body: gene body; IGR: Intergenic Region; island: CpG island; shore: 0-2kb up- or downstream from CpG island; shelf: 2-4kb up- or downstream from CpG island; open sea: > 4 kb up- or downstream from CpG island. Annotation based on UCSC (genome.ucsc.edu) GRCh37/hg19 reference.
