# Supplementary figures and images for: Differential DNA methylation at birth associated with mental disorder in individuals with 22q11.2 deletion syndrome
Source: Transl Psychiatry. 2017 Aug 29;7(8):e1221–. doi: 10.1038/tp.2017.181 (PMC5611746; doi:10.1038/tp.2017.181)

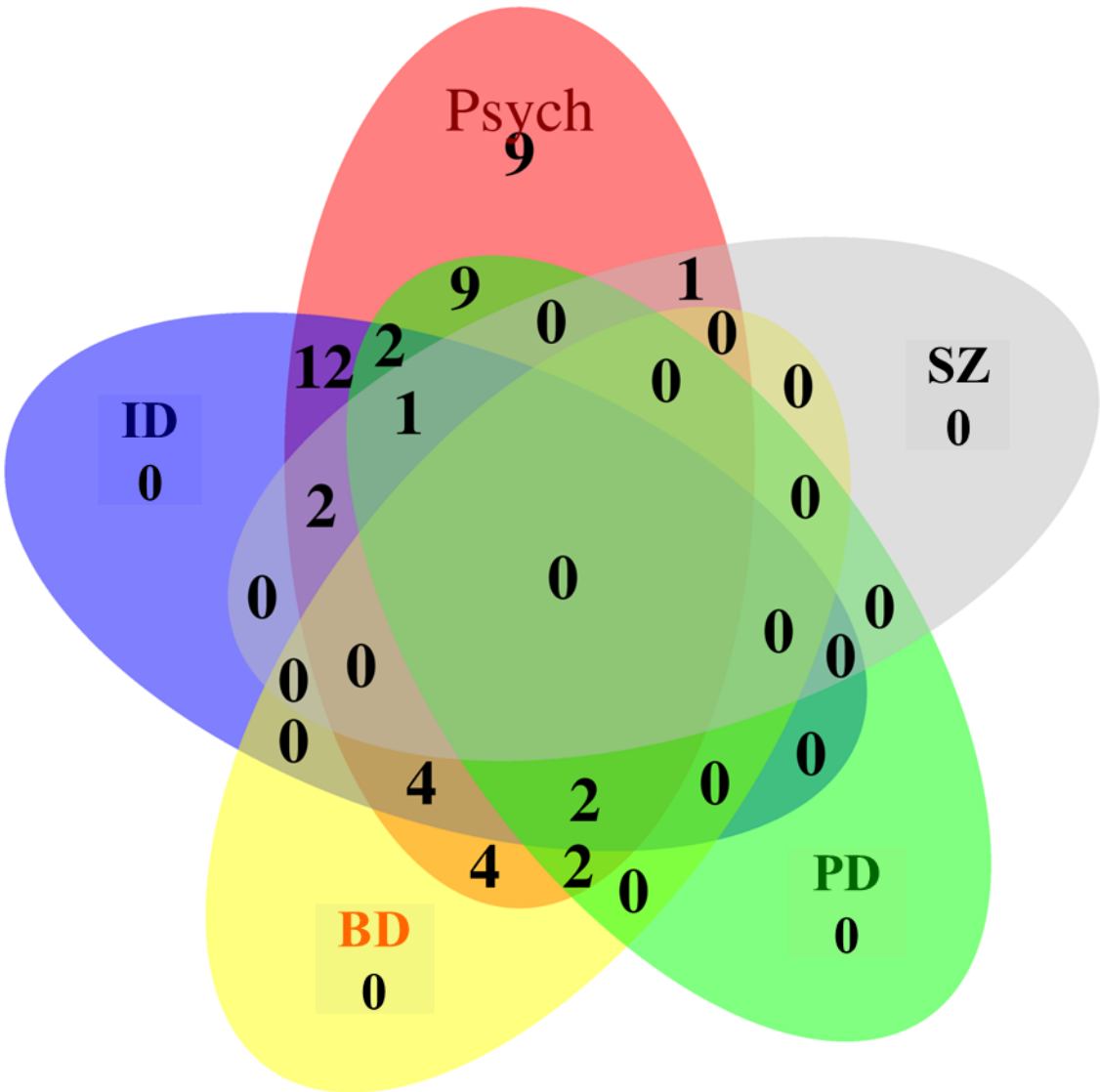

Supplement: Supplementary Figure 1 [file tp2017181x5.pdf]

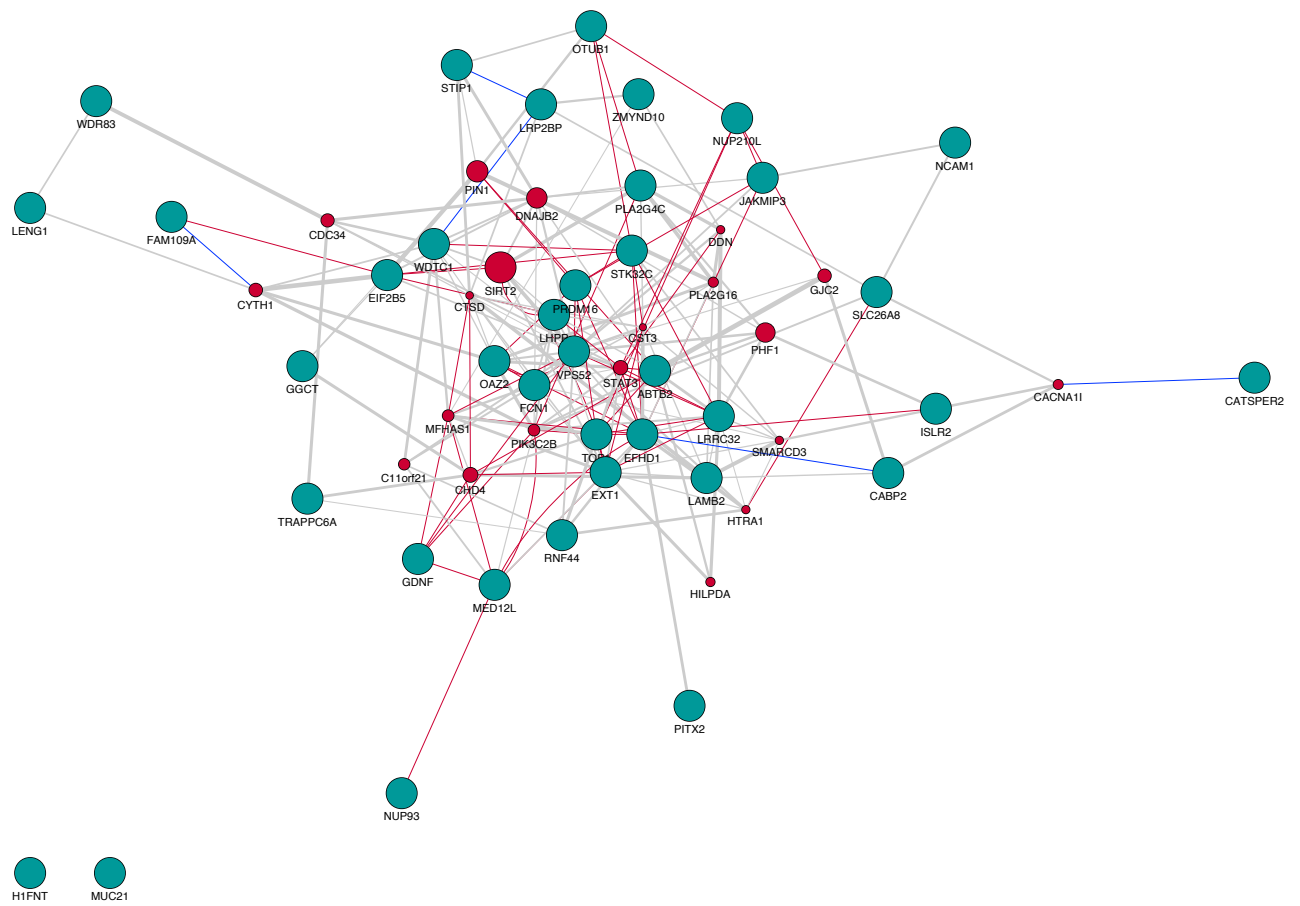

Supplement: Supplementary Figure 2 [file tp2017181x6.pdf]

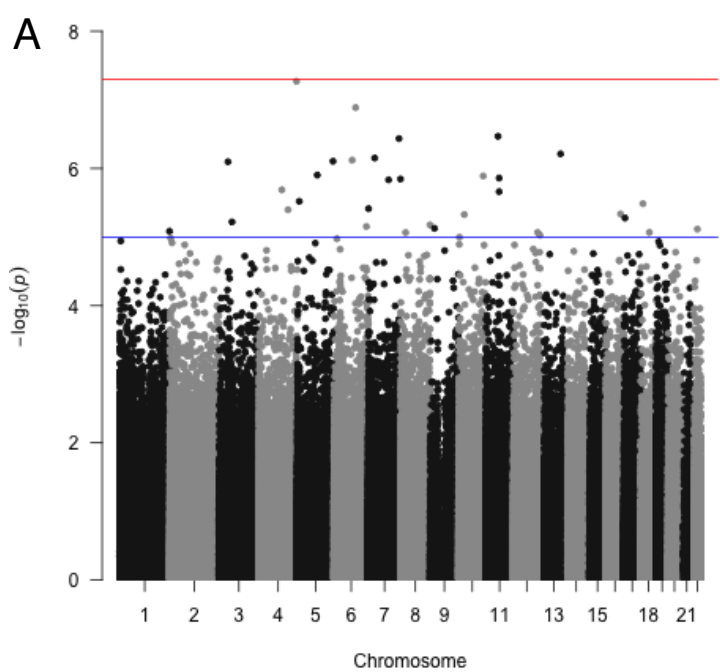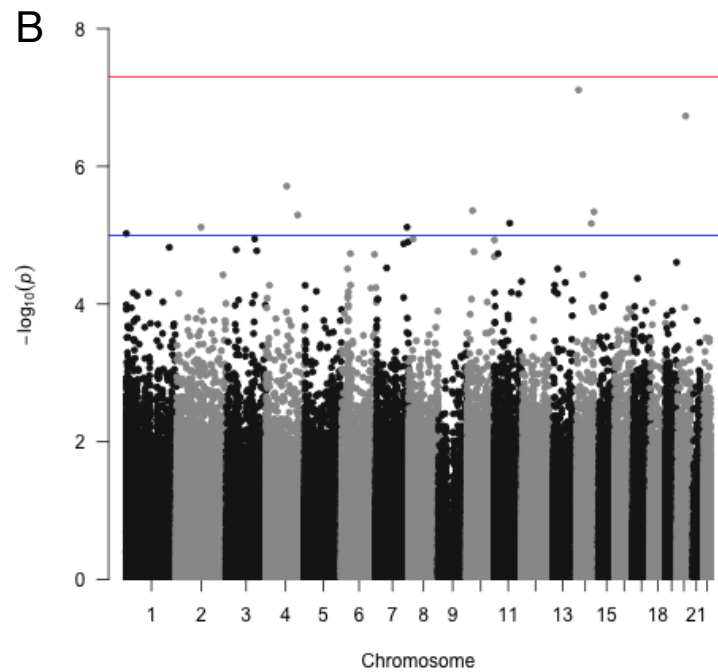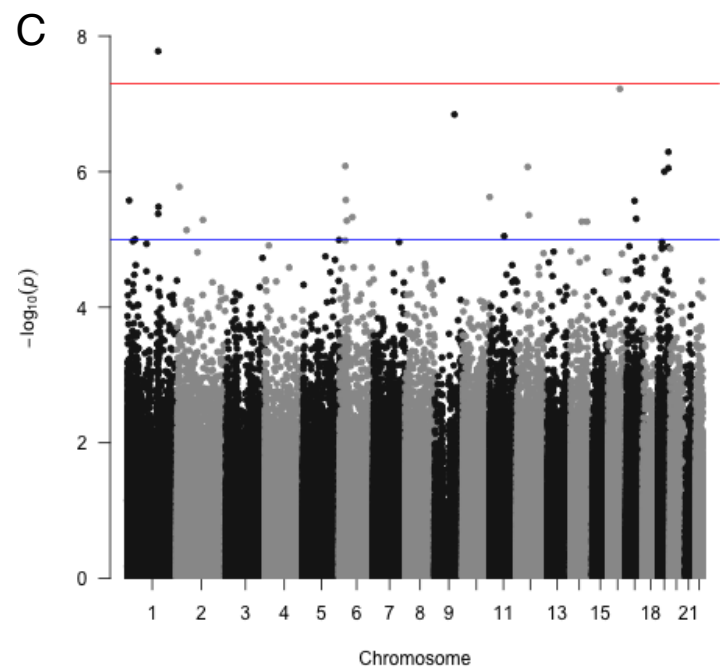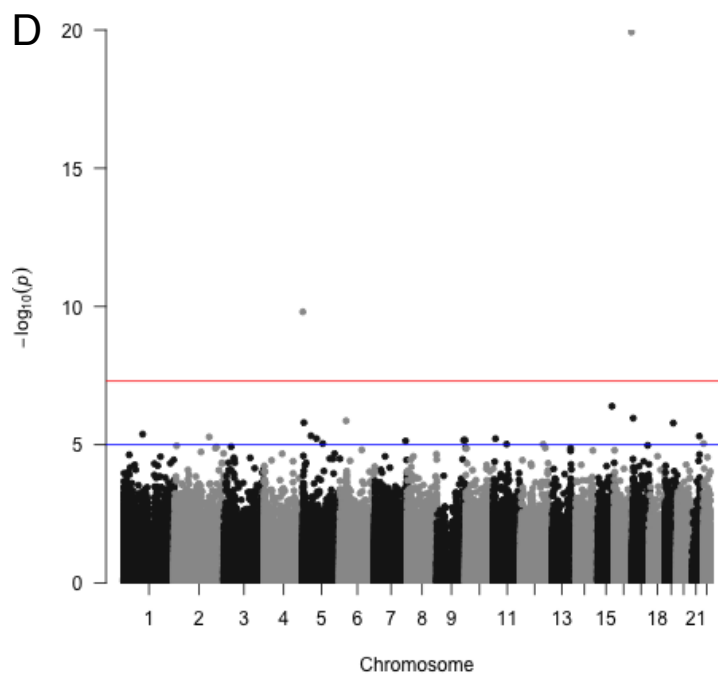

Supplement: Supplementary Figure 3 [file tp2017181x7.pdf]

**A**

cg04355077, NOSIP

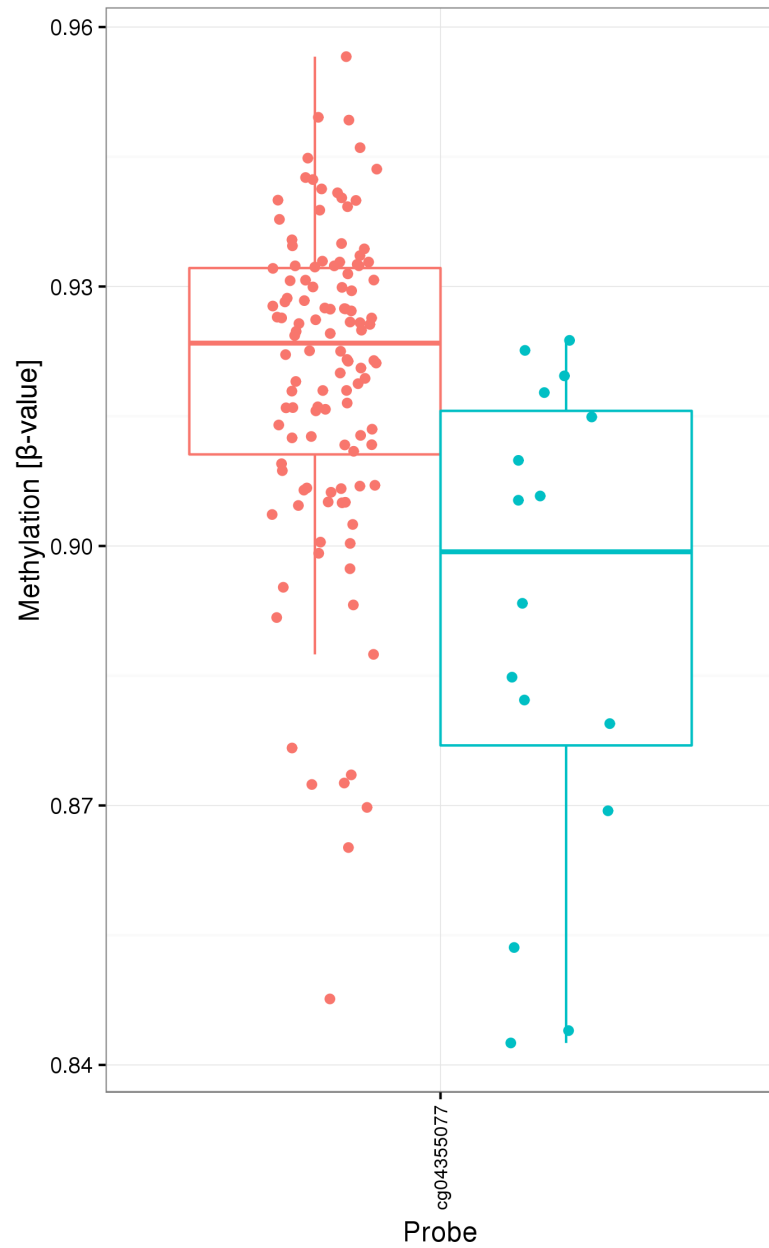**B**

cg21292909, NOSIP

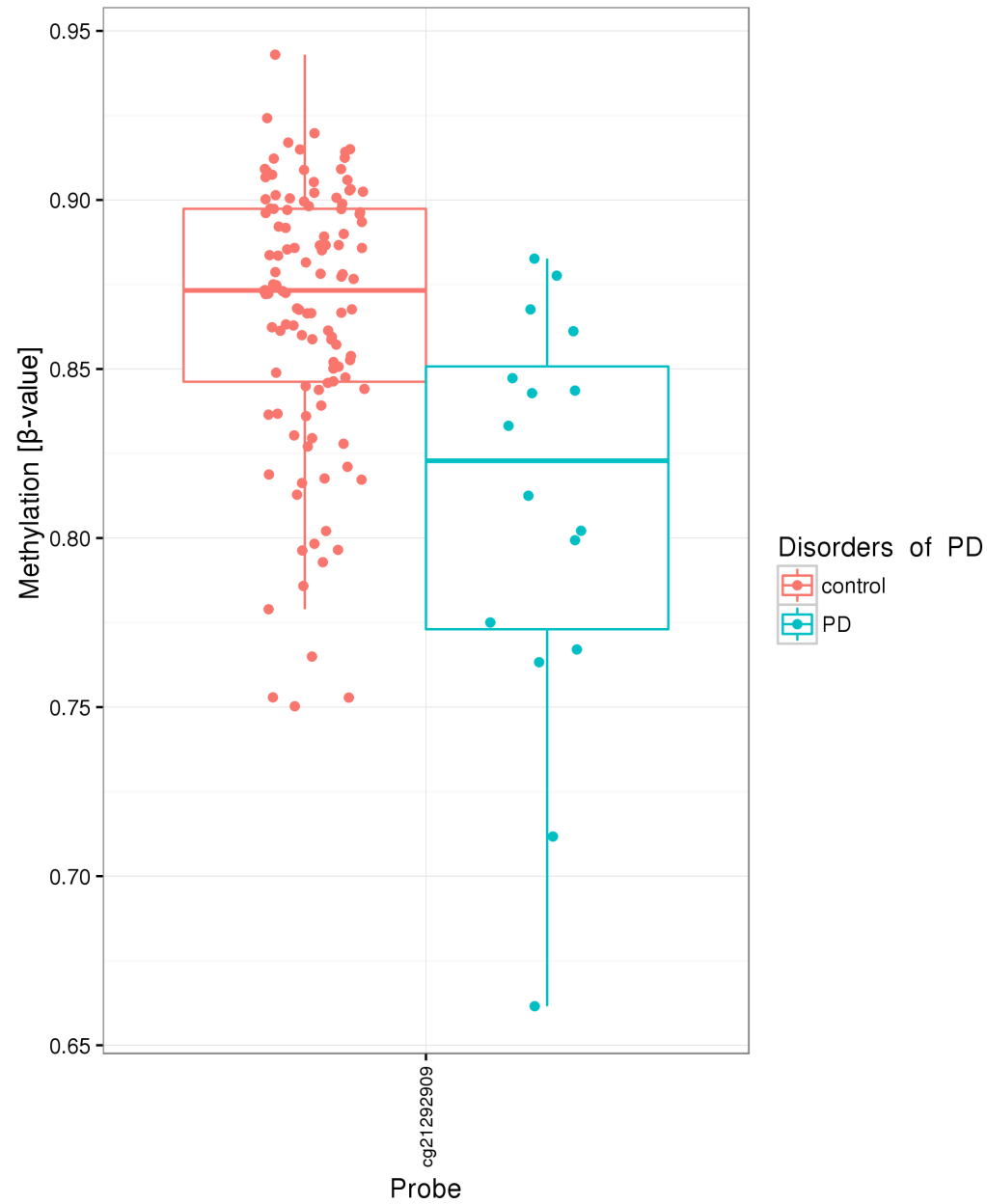

Supplement: Supplementary Figure 4 [file tp2017181x8.pdf]

P-value < 0.00001

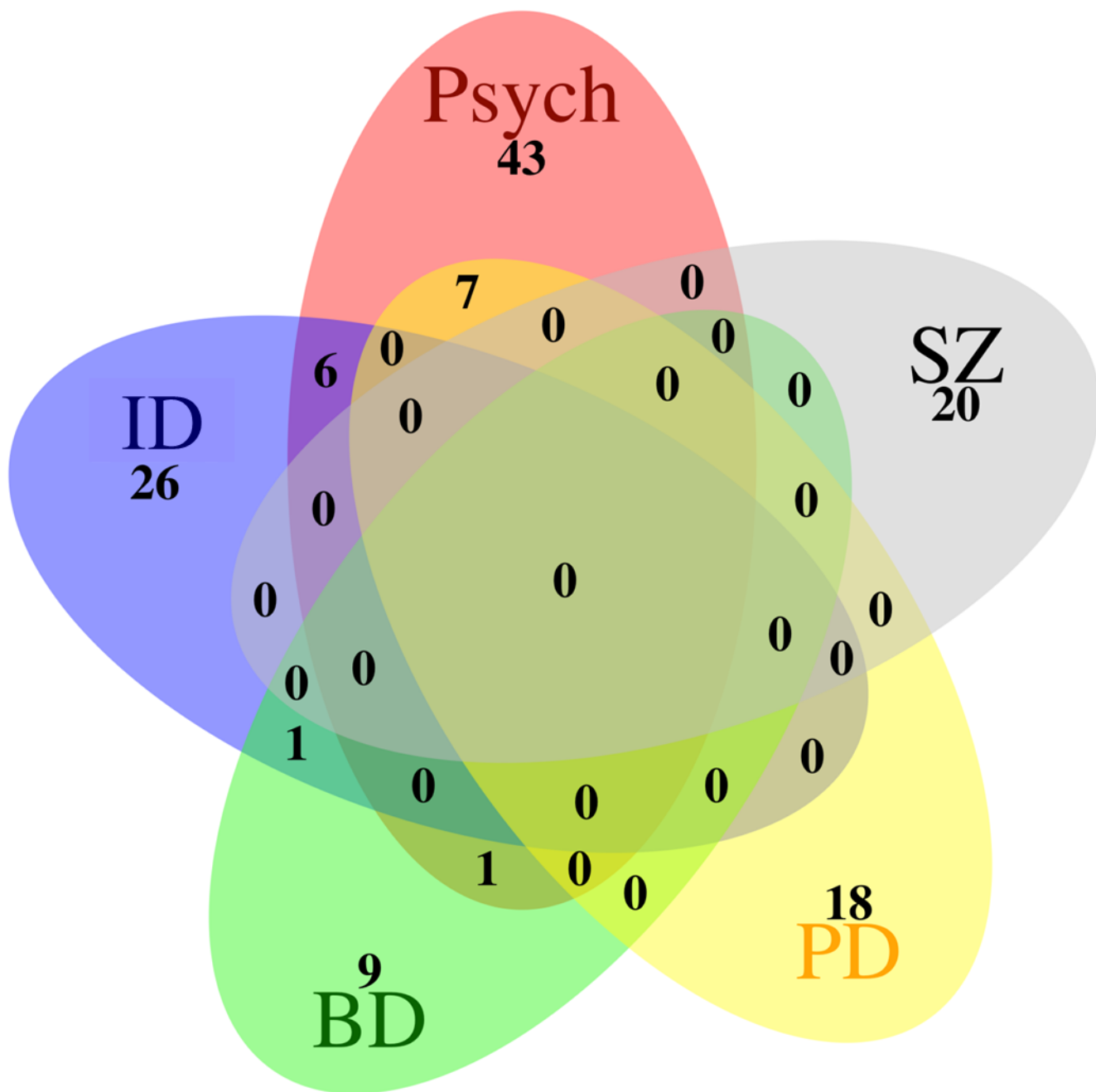

Supplement: Supplementary Figure 5 [file tp2017181x9.pdf]

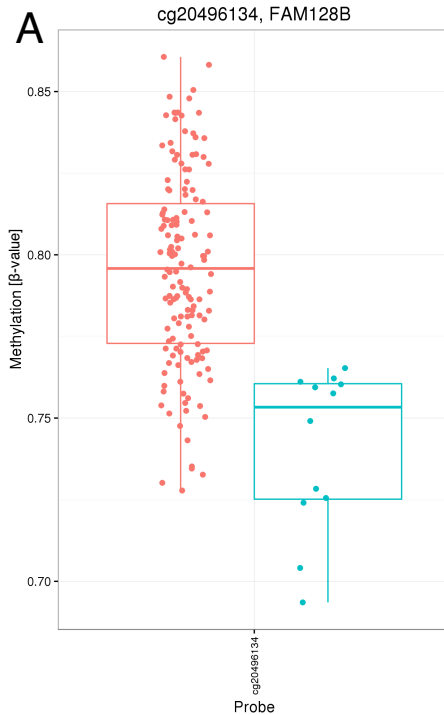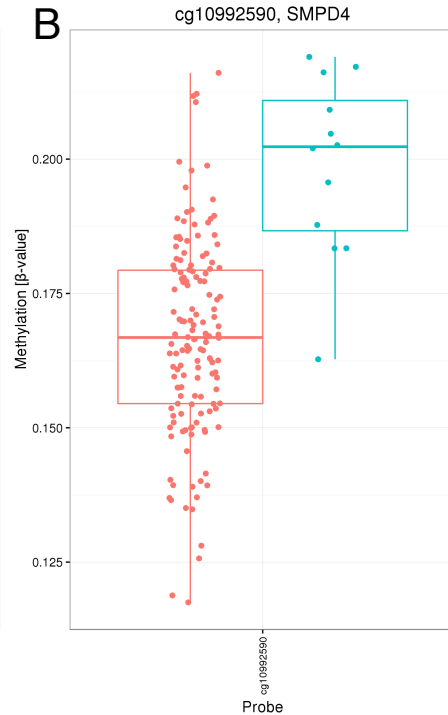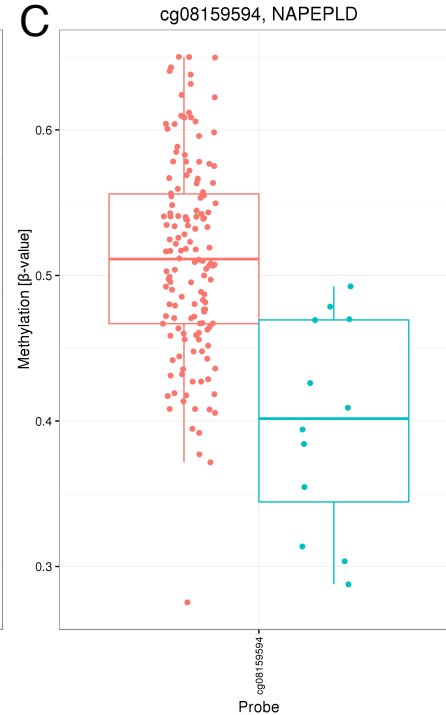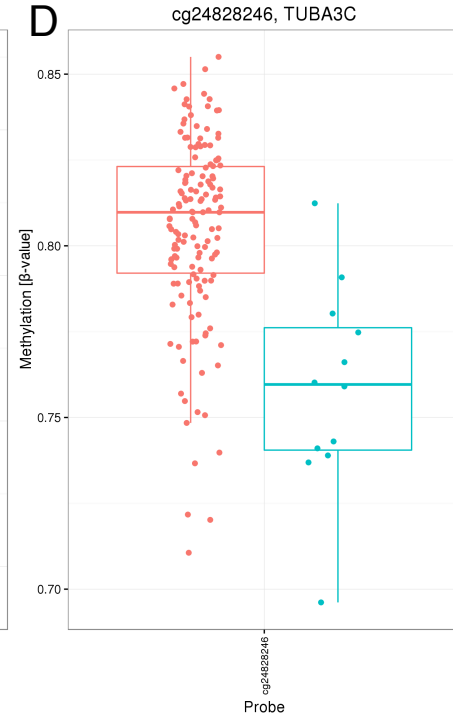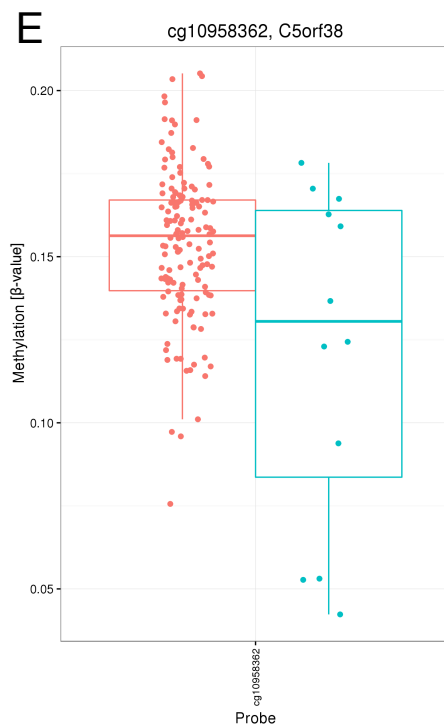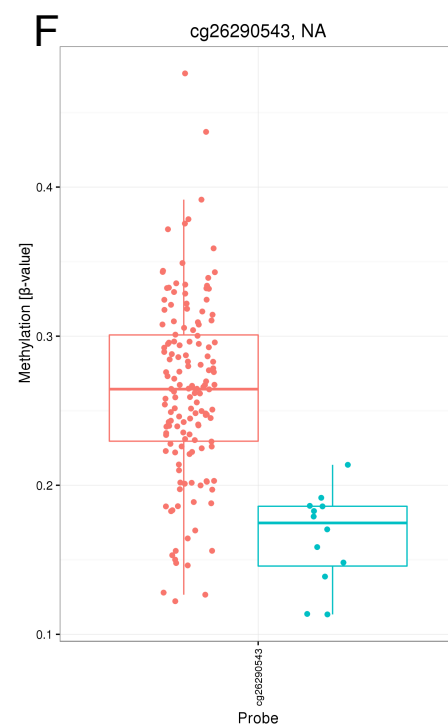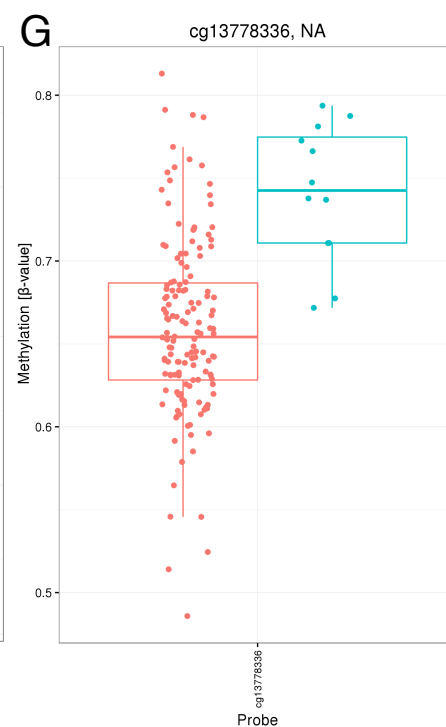

Supplement: Supplementary Figure 6 [file tp2017181x10.pdf]
